# 3D Chiral Self‐Assembling Matrixes for Regulating Polarization of Macrophages and Enhance Repair of Myocardial Infarction

**DOI:** 10.1002/advs.202304627

**Published:** 2023-09-28

**Authors:** Lei Yang, Li Yang, Kongli Lu, Nan Su, Xueqin Li, Shuoxiang Guo, Song Xue, Feng Lian, Chuanliang Feng

**Affiliations:** ^1^ Department of Cardiovascular Surgery Renji Hospital School of Medicine Shanghai Jiao Tong University 160 Pujian Road Shanghai 200127 P. R. China; ^2^ State Key Lab of Metal Matrix Composites School of Materials Science and Engineering Shanghai Jiao Tong University 800 Dongchuan Road Shanghai 200240 P. R. China

**Keywords:** chiral self‐assembling matrixes, immunological response, macrophages, myocardial infarction

## Abstract

The regulation of inflammatory response at the site of injury and macrophage immunotherapy is critical for tissue repair. Chiral self‐assemblies are one of the most ubiquitous life cues, which is closely related to biological functions, life processes, and even the pathogenesis of diseases. However, the role of supramolecular chiral self‐assemblies in the regulation of immune functions in the internal environment of tissues has not been fully explored yet. Herein, 3D supramolecular chiral self‐assembling matrixes are prepared to regulate the polarization of macrophages and further enhance the repair of myocardial infarction (MI). Experiments studies show that *M*‐type (left‐handed) self‐assembling matrixes significantly inhibit inflammation and promote damaged myocardium repair by upregulating M2 macrophage polarization and downstream immune signaling compared with *P*‐type (right‐handed), and *R*‐type (non‐chirality) self‐assembling matrixes. Classical molecular dynamics (MD) simulation demonstrates that *M*‐type self‐assembling matrixes display higher stereo‐affinity to cellular binding, which enhances the clustering of mechanosensitive integrin β1 (Itgβ1) and activates focal adhesion kinase (FAK) and Rho‐associated protein kinase (ROCK), as well as downstream PI3K/Akt1/mTOR signaling axes to promote M2 polarization. This study of designing a 3D chiral self‐assembling matrixes microenvironment suitable for regulating the polarization of macrophages will provide devise basis for immunotherapy with biomimetic materials.

## Graphical abstract

A study of designing 3D chiral self‐assembling matrixes suitable for regulating the polarization of macrophages to inhibit inflammation and promote the repair of damaged myocardium.

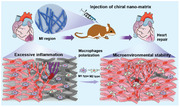

## 1 Introduction

Myocardial infarction (MI) usually occurs in coronary artery occlusion, resulting in myocardial necrosis caused by persistent ischemia and hypoxia, which presents high morbidity and mortality.^[^ ^1^ ^]^ After MI, innate immunity is rapidly activated to produce a strong and transient inflammatory response, clearing the infarct region of dead cells and extracellular matrix debris, followed by a proliferation phase. During the proliferative phase, monocytes and macrophage subpopulations secrete growth factors that recruit and activate mesenchymal repair cells (mainly myoblasts and blood vessel cells). Myofibroblasts secrete large amounts of extracellular matrix proteins, which preserve the intact structure of the left ventricle and ultimately lead to the formation of scar collagen fibers. Repair of damaged tissue relies on timely suppression of inflammation, and inadequate suppression of cardiac inflammation can lead to catastrophic outcomes, resulting in loss of cardiomyocytes, suppression of systolic function, enlargement of the ventricles, loss of ventricular wall integrity, and rupture of the heart.^[^ ^2^ ^]^ The efficacy of traditional cardiac repairing strategies including drug and cell therapies for MI treatment is greatly limited by the cardiac microenvironment, which might lead to cardiac remodeling after MI and ultimately result in heart failure.^[^ ^3^ ^]^ Macrophage immunotherapy targeting the MI microenvironment is an emerging treatment approach that modulates the immune system to promote the repair of MI.^[^ ^4^ ^]^ Macrophages are one of the main responder cells of the immune system, infiltrating the infarcted myocardium for repairing MI, which can be roughly divided into early pro‐inflammatory macrophage phenotype I (M1) and later anti‐inflammatory macrophage phenotype II (M2).^[^ ^5^ ^]^ Macrophage behavior of various tissues during healing is closely associated with several biochemical and biophysical properties of the extracellular matrix (ECM), including chemical species, supramolecular self‐assemblies, mechanical, and chiral properties.^[^ ^6^ ^]^ Among these, chiral supramolecular self‐assemblies as basic structural units of living organisms are crucial for the good implementation of sophisticated functions involved in the life process.^[^ ^7^ ^]^ However, the strategy by which biologically bionic ECM based on 3D chiral supramolecular self‐assemblies manipulates the regulation of macrophages’ immune response has rarely been explored.

The ECM network plays a critical role in cardiac homeostasis by providing structural support, facilitating force transmission, and transducing key signals to cardiomyocytes, vascular cells, and interstitial cells.^[^ ^8^ ^]^ The insoluble matrix components in ECM could offer various 3D biophysical cues to the tissue microenvironment at the nanometer to micron scales.^[^ ^9^ ^]^ Macrophages can dynamically interact with ECM‐mediated microenvironment, regulating their adhesion and polarization, mediated by the dynamic binding of integrin and ligand motifs in vivo, such as the RGD tripeptide sequence (Arg‐Gly‐Asp).^[^ ^10^ ^]^ RGD plays an important role in the innate immune system in the adhesion of myeloid cells such as granulocytes and macrophages. Both soluble and surface‐functionalized RGD polypeptides bind to myeloid cells via integrin.^[^ ^11^ ^]^ Blocking integrin binding with soluble RGD polypeptide significantly reduces TNFα and IL‐6 production.^[^ ^12^ ^]^ Macrophages also exhibit altered immunomodulatory effects due to RGD interactions, including changes in phenotype, cytokine production, and phagocytosis.^[^ ^5^, ^13^ ^]^ Accordingly, exploring the interactions between RGD peptides and biomimetic ECM to regulate macrophage polarization and then obtain the expected host immune response is becoming a novel method for repairing MI.

Supramolecular chiral self‐assemblies (e.g., double helix in DNA, α‐helix, and β‐sheet in proteins) arising from the asymmetric spatial stacking of molecular blockings are involved in various essential biological processes, including the maintenance of cell morphology and functional changes in the tissue microenvironment.^[^ ^14^ ^]^ Pioneering research revealed that molecular chirality modified on traditional 2D matrixes could affect cellular asymmetrical motions that result in the different behaviors of cell morphology.^[^ ^15^ ^]^ However, exploring how artificial 3D supramolecular chiral matrixes influence cell behaviors in the ECM is particularly important since 3D supramolecular chiral matrixes extensively exist in biophysical ECM, which is required for relevant biological events.^[^ ^7^, ^16^ ^]^ Recently, the effect of the physical properties of ECM (such as tension and mechanical properties) on the phenotype and behavior of macrophages has received extensive attention.^[^ ^17^ ^]^ However, the potential importance of 3D chiral supramolecular self‐assemblies to affect the adsorption of adhesion proteins or peptides on the surface of materials and thus on the behavior of the polarization of macrophages is seldom explored.

**Scheme 1:**
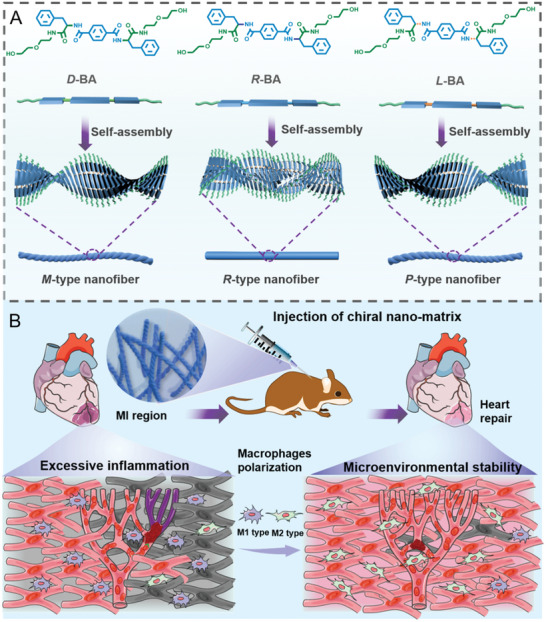
A) A schematic representation of biologically 3D self‐assembling matrixes with tunable supramolecular chirality. B) Schematic diagram showing 3D chiral self‐assembling matrixes regulating the polarization of macrophages and enhancing repair of myocardial infarction (MI).

Herein, enantiomeric *L*/*D*‐phenylalanine gelators (*L*‐BA and *D*‐BA) and racemic *DL*‐phenylalanine gelators (*R*‐BA) are designed to construct cell‐supporting 3D chiral supramolecular nanofibers matrixes *by* a self‐assembly strategy, respectively, to regulating macrophage phenotype in the MI region, thereby facilitating MI repair (**Scheme** **1**). Experiment studies investigate that *M*‐type supramolecular nanofibers matrixes significantly inhibit inflammation and promote damaged myocardium repair by upregulating M2 macrophage polarization and downstream immune signaling in contrast to corresponding *P*‐type and *R*‐type supramolecular nanofibers matrixes. The initial enantioselective mechanism between different chiral molecules and peptides was theoretically performed by classical molecular dynamics (MD) simulation. This work unveils the interaction between the biomimetic 3D supramolecular chiral matrixes and the macrophages’ immune system, which inspires alternatively sophisticated for developing immunotherapeutics.

## 2 Results and Discussion

### 2.1 Preparation, Morphology, and Supramolecular Chirality of 3D Self‐Assembling Matrixes

**Figure 1:**
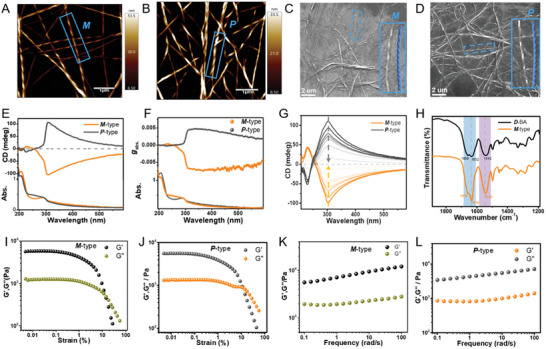
AFM images of helical nanofibers: A) left‐handed (*M‐*type) helical nanofibers obtained from the self‐assembly of *D*‐BA and B) right‐handed (*P‐*type) helical nanofibers obtained from the self‐assembly of *L*‐BA. SEM images of helical nanofibers: C) *M‐*type helical nanofibers obtained from the self‐assembly of *D*‐BA and D) *P‐*type helical nanofibers obtained from the self‐assembly of *L*‐BA. E) Circular dichroism (CD) spectroscopy of *M‐*type and *P‐*type self‐assembling matrixes. F) Corresponding *g*_abs_ and UV–vis spectroscopy of *M‐*type and *P‐*type self‐assembling matrixes. G) Temperature‐dependent CD spectroscopy of *M‐*type and *P‐*type self‐assembling matrixes. H) FTIR spectra of *D*‐BA and *M*‐type self‐assembling matrixes. I, J) Strain‐dependent oscillatory shear rheology of *M*‐type and *P*‐type hydrogels, respectively. K, L) Dynamic frequency sweep of *M*‐type and *P*‐type self‐assembling matrixes, respectively.

Enantiomeric *D*/*L*‐phenylalanine gelator (*D*‐BA and *L*‐BA) and racemic *DL*‐phenylalanine gelator (*R*‐BA) are employed to mimic the biophysical environment of natural ECM (synthesis and characterization of gelators see Schemes S1,S2 and Figures S1–S6, Supporting Information). *D*‐BA and *L*‐BA molecules can self‐assemble into *M*‐type nanofibers and *P*‐type nanofibers, respectively, while *R*‐BA molecules can self‐assemble into nanofibers without distinct chirality. These high‐aspect‐ratio nanofibers template through hydrogen bonding along the long axis of nanofibers and further physically entangle to self‐supportive 3D hydrogels networks *by* a supramolecular gelation process (Scheme 1A).^[^ ^18^ ^]^ The critical gel concentration (CGC) for *L*‐BA, *D*‐BA, and *R*‐BA is 1.0 wt.%, and hydrogel formation was judged by the conventional tube inversion method (Figure S7 and Table S1, Supporting Information). The water content of these three hydrogels was measured by the method of drying and weighing was almost above 98% (Figure S8A, Supporting Information). Thermal analysis results demonstrated that the denaturation temperatures of *L*‐BA, *D*‐BA, and *R*‐BA were all higher than 37 °C, indicating their good thermal stabilities. (Figure S8B, Supporting Information). Atomic force microscopy (AFM) and scanning electron microscopy (SEM) were used to investigate the morphology of the supramolecular hydrogels. The self‐assembly of *D*‐BA formed exclusively left‐handed (*M*‐type) helical nanofibers, while the self‐assembly of *L*‐BA produced uniform right‐handed (*P*‐type) helical nanofibers. We have made distribution statistics of the diameter (100 ± 28 nm) and helical pitch (500 ± 20 nm) of these fiber nanostructures, they are almost the same except presented different chiral nanostructures (**Figure** **1**A–D; Figure S9, Supporting Information). Additionally, *R*‐BA was self‐assembled into uniform nanofibers without distinct chiral features (Figures S10,S11, Supporting Information).

The supramolecular chirality of the *M*‐type, *P*‐type, and *R*‐type supramolecular hydrogels was investigated *by* circular dichroism (CD) spectroscopy, respectively. The CD spectrum of *M*‐type hydrogels showed a relatively weak positive Cotton effect at 230 nm and a strong negative Cotton effect at 306 nm (Figure 1E, yellow curve), whereas *P*‐type hydrogels showed the mirror‐imaged CD spectrum of *M*‐type hydrogels (Figure 1E, gray curve). The *M*‐type and *P*‐type hydrogels showed the absorptive dissymmetry factor *g*_abs_, which is defined by the ratio of CD intensity to the corresponding absorption (shown in Supporting Information), were approximately equal in intensity and handedness opposite in chirality (Figure 1F), which indicated that the molecular chirality of the gelators is well transferred to the supramolecular chirality.^[^ ^14^, ^18^ ^]^ With the increasing testing temperature, the intensity of the CD signals for both *M*‐type and *P*‐type self‐assembling hydrogels gradually decreased due to disassembly by heating. At ≈95 °C, the CD signals at 230 and 306 nm both almost disappeared suggesting the disintegration of *M*‐type and *P*‐type self‐assembled nanofibers, respectively. Two new weak CD peaks at 218 and 232 nm were ascribed to the molecular chirality from phenylalanine in *D*‐BA and *L*‐BA, respectively (Figure 1G). These data demonstrate that the chirality of the 3D hydrogels can be attributed to the self‐assembled fibrous aggregates and not to the individual monomers.^[^ ^7^, ^18^ ^]^ In addition, no CD signals existed for *R*‐type hydrogels due to their natural racemic characteristics (Figure S12, Supporting Information).

### 2.2 Mechanism and Mechanical Properties of 3D Self‐Assembling Matrixes

The underlying mechanism of the self‐assembly was further confirmed by fourier transform infrared (FTIR) spectroscopy measurement. For *D*‐BA as an example, the monomeric state of C═O stretching vibration of the peripheral amide and the central amide close to the benzene core at 1674 and 1652 cm^−1^ shifted to 1658 and 1631 cm^−1^, respectively, after self‐assembly. FTIR results suggest hydrogen bonding interactions are stronger due to the intramolecular H─bonds with a small bond length formed from the amino acid diamides (Figure 1H). The mechanical properties of supramolecular self‐assembling matrixes (*M*‐type, *P*‐type, and *R*‐type hydrogels) were measured by a rotary rheometer with dynamic frequency sweep at a strain of 0.01%−100%, respectively, the values of elastic modulus (G′) of three hydrogels were higher than those of the viscous modulus values (G″), indicating three supramolecular self‐assembling matrixes formed solid hydrogels. (Figure 1I–L; Figure S13, Supporting Information). Hence, these supramolecular self‐assembling matrixes provide an adequate platform to study the effect of chiral nanostructure on immunological response.

### 2.3 Effect of 3D Chiral Self‐Assembling Matrixes on Cytocompatibility

Supramolecular self‐assembling chiral nanofibers have good biocompatibility in cell adhesion and proliferation as demonstrated in our previous works.^[^ ^7^, ^18^ ^]^ To verify the heterogenic of different 3D chiral self‐assembling matrixes in cardiac cells, we first observed the chiral self‐assembling matrixes have cytocompatibility in cell adhesion and proliferation. We cultured macrophages, fibroblasts, and cardiomyocytes in *M*‐type, *R*‐type, and *P*‐type self‐assembling matrixes for 24, 48, and 72 h, respectively. A cell counting kit‐8 (CCK‐8) assay showed different activities of macrophages, fibroblasts, and cardiomyocytes in different cultural chiral self‐assembling matrixes environments, separately. Compared with the *P*‐type and *R*‐type self‐assembling matrixes, the *M*‐type self‐assembling matrixes reduced the apoptosis rate of macrophages, fibroblasts, and cardiomyocytes after 72 h of culture (Figure S14, Supporting Information). These results indicate that *M*‐type self‐assembling matrixes have better cytocompatibility compared to *P*‐type and *R*‐type self‐assembling matrixes.

### 2.4 Effect of 3D Chiral Self‐Assembling Matrixes on Polarization of Macrophages

**Figure 2:**
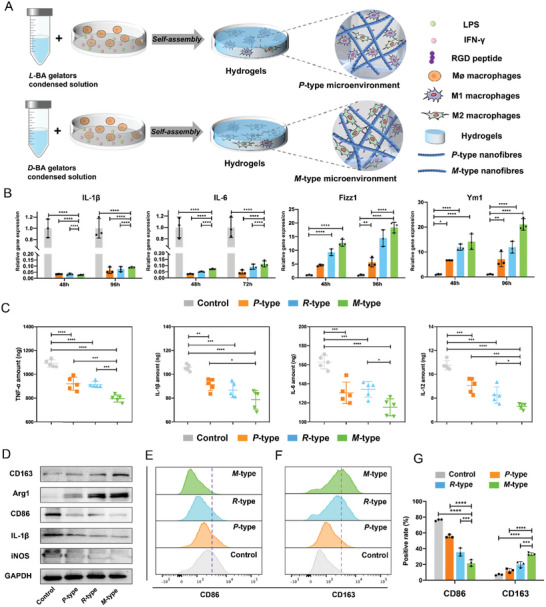
A) Schematic depicting the process of chirality‐dependent macrophage induction of polarization. B) RT‐qPCR results showed the expression of M1‐related proinflammatory factors IL‐1β and IL‐6 and anti‐inflammatory factor Ym1 and M2 marker Fizz1 in *the Control* group, *P*‐type, *R*‐type, and *M*‐type matrixes. C) ELISA results showed that M1‐related pro‐inflammatory factors TNF‐α, IL‐1β, IL‐6, and IL‐12 were expressed in *Control* group, *P*‐type, *R*‐type, and *M*‐type matrixes. D) Western blot showed the expression of M2 surface markers in *M*‐type, *P*‐type and *R*‐type self‐assembled matrixes. E, F) The expression of CD206 and CD86 in *Control* group, *P*‐type, *R*‐type and *M*‐type matrixes was detected by flow cytometry. G) Quantitative analysis showed the results of flow cytometry.

The inflammatory response in the early stage of trauma is critical for healing the damaged tissue.^[^ ^5^, ^19^ ^]^ In the initial stage of trauma development, naive macrophages (Mϕ) are responsible for phagocytosis, releasing pro‐inflammatory mediators, clearing senescent cells, and participating in tissue repair processes. Still, during the process of the damaged healing, macrophages initially present a classically activated M1 phenotype, high surface markers or intracellular iNOS can be used to identify M1 macrophages that release inflammatory cytokines (such as IL‐1β, IL‐6, IL‐12, and TNFα).^[^ ^5^, ^20^ ^]^ Following the inflammatory phase, macrophages differentiate into alternatively activated M2 types. M2 macrophages can secrete anti‐inflammatory mediators such as surface markers of scavenger receptors, IL‐10, intracellular Arginase‐1, found in inflammatory zone 1(Fizz1), and Chitinase‐like 3 (Chil3/Ym1) expression. In this research, novel 3D supramolecular hydrogel systems with chiral nanofibrous structures were developed for studying supramolecular chirality‐dependent immunological responses (**Figure** **2**A). To reveal supramolecular chirality regulating the polarization of macrophages, RT‐qPCR results showed the expression of the M1‐related inflammatory genes IL‐1β, IL‐6 (Figure 2B), iNOS, and TNF‐α (Figure S15, Supporting Information) in *M*‐type matrixes were significantly lower than those in *P*‐type and *R*‐type matrixes, respectively. The expression of M2‐related genes including Ym1, Fizz1 (Figure 2B), and TLR9 (Figure S15, Supporting Information) in *M*‐type matrixes were significantly increased compared to *P*‐type and *R*‐type matrixes, respectively. We also quantified four key pro‐inflammatory cytokines released by macrophages in the control group and different supramolecular self‐assembling matrixes groups after 48 h of culture, respectively. Herein, lipopolysaccharide (LPS) and interferon γ (IFN‐γ) induced macrophages as a positive control group (denoted as *Control*). The levels of secretion of TNF‐α, IL‐6, IL‐12, and IL‐1β in the *Control* were significantly more than those in the *M*‐type, *R*‐type, and *P*‐type self‐assembling matrixes groups (P < 0.001) (Figure 2C). In addition, the expression of TNF‐α, IL‐6, IL‐12, and IL‐1β in *M*‐type self‐assembling matrixes was significantly decreased compared to those in *R*‐type and *P*‐type self‐assembling matrixes. These results indicated that macrophages recognized the difference of chiral self‐assembling matrixes thus polarized to divergent phenotypes, more importantly, our findings showed the effect of *M*‐type chirality is stronger than that of *P*‐type chirality.

The pro‐inflammatory macrophages (M1 macrophages) express high levels of iNOS while the anti‐inflammatory macrophages (M2 macrophages) express high levels of CD163.^[^ ^5^, ^21^ ^]^ Immunofluorescence was used to analyze the changes in cell phenotype and the expression of membrane protein after the polarization of macrophages. Immunofluorescence staining showed the number of macrophages with the M1 surface marker iNOS in the *Control* group was much higher than those in the three supramolecular self‐assembling matrixes groups. In contrast, the number of macrophages with the M2 surface marker CD163 in three supramolecular self‐assembling matrixes groups was much higher than those in the *Control* group (Figure S16, Supporting Information). Western blot proved that the proportion of M2 surface markers in *M*‐type self‐assembling matrixes was much higher than that in the *R*‐type and *P*‐type self‐assembling matrixes, respectively (Figure 2D). Moreover, the flow cytometry assay confirmed that the expression of CD163 and CD206 were significantly upregulated in *M*‐type self‐assembling matrixes than in *R*‐type and *P*‐type self‐assembling matrixes. (Figure 2F; Figure S17, Supporting Information). The expression of CD86 in *M*‐type self‐assembling matrixes was significantly down‐regulated compared with that in *R*‐type and *P*‐type self‐assembling matrixes (Figure 2E,F). The above data indicate the *M*‐type self‐assembling matrixes can up‐regulate M2‐related membrane protein expression and promote M2 polarization in macrophages more than the *P*‐type and *R*‐type matrixes.

**Figure 3:**
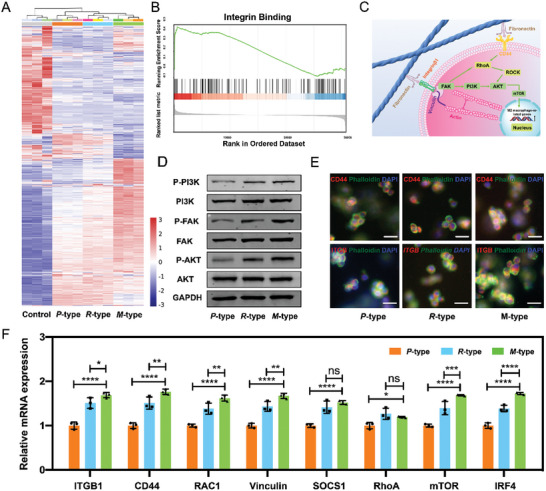
A) A heatmap representing major altered genes in three datasets of macrophages treated with *P*‐type, *R*‐type, and *M*‐type matrixes. Macrophages with LPS+IFN‐γ treatment were used as a negative control. The color bar indicates normalized z‐score intensity‐based absolute quantification. B) Gene enrichment analysis showed that *M*‐type matrixes were enriched in integrin binding‐related genes related to M2 polarization. C) Schematic diagram of specific molecular signal transduction in M2 macrophages induced by chiral matrixes. D) Western blotting analysis revealed the expression of mechanoinductive and transducing proteins (P‐PI3K, P‐FAK, P‐AKT) in *M*‐type, *P*‐type and *R*‐type matrixes. E) Immunofluorescence analysis showed the expression of CD44 and ITGB in *M*‐type, *P*‐type and *R*‐type matrixes, scale :5 µm. F) Quantitative RT‐qPCR analysis revealed the expression of typical M2‐related genes and mechanism pathway genes in macrophages in *M*‐type, *P*‐type and *R*‐type matrixes.

Reduced release of pro‐inflammatory cytokines can be regarded as one of the types of evidence for the phenotypic transformation of macrophages.^[^ ^5a^ ^]^ To further investigate the difference in the immune response produced by the chirality‐dependent macrophage polarization, gene expression profiling analysis was performed. Cluster analysis displayed that macrophages had overexpressed and inhibitory genes in all groups (**Figure** **3**A). The expression of adhesion genes and M2‐related genes in *M*‐type, *R*‐type, and *P*‐type self‐assembling matrixes were up‐regulated while the expression of most of the M1‐related genes was down‐regulated. Compared with the *P*‐type and *R*‐type self‐assembling matrixes, the *M*‐type self‐assembling matrixes had a greater degree of genetic difference (Figure S18, Supporting Information). The results of gene ontology analysis and pathway enrichment analysis showed that the TNF signaling pathway, mTOR signaling pathway, NF‐kappa B signaling pathway, and Cytokine‐Cytokine receptor interaction signaling pathway were activated (Figures S19–S22, Supporting Information). Gene enrichment analysis presented that integrin binding‐related genes associated with M2 polarization were enriched in *M*‐type self‐assembling matrixes (Figure 3B), which was consistent with the results in the above gene expression profile analysis. The volcano map showed that compared with the control group, the expression of ARG1 in *P*‐type, *R*‐type, and *M*‐type groups was gradually up‐regulated. The Venn map showed *M*‐type, *P*‐type, and *R*‐type were all different compared with the control group, among that the *M*‐ type had the largest number of differential genes (Figure S23 Supporting Information). These results suggest that the macrophages in the *M*‐type self‐assembling matrixes had a stronger immune activation effect than those in the *R*‐type and *P*‐type self‐assembling matrixes.

Recent research has reported ECM assembling and remodeling, cytoskeletal remodeling, and tissue repair were closely related to the polarization of M2 macrophages,^[^ ^5^, ^21^ ^]^ which inspired us to explore the underlying molecular mechanism of macrophage polarization induced by supramolecular chirality. We referred to the M2 polarization signaling pathway of macrophages and found most likely signaling pathway involved in adhesion‐induced polarization might be the PI3K/AKT/mTOR signal axis.^[^ ^22^ ^]^ Western blotting results showed the phosphorylation of PI3K, FAK, and Akt1 was found to be significantly higher in the *M*‐type self‐assembling matrixes than in *P*‐type and *R*‐type self‐assembling matrixes (Figure 3D). Immunofluorescence staining exhibited that the expression of integrin β1 (Itgβ1) and CD44 were up‐regulated in the *P*‐type, *M*‐type, and *R*‐type self‐assembling matrixes, respectively. While the higher expression of Itgβ1 and CD44 in *M*‐type matrixes than in *P*‐type and *R*‐type matrixes (Figure 3E) indicated that more focal adhesions of cellular were formed in the *M*‐type matrixes.^[^ ^23^ ^]^ Therefore, we proposed 3D chiral self‐assembling matrixes microenvironment potentially affects FAK/Rho/ROCK signaling pathways activated by integrin and Rho/ROCK signaling pathways activated by CD44, which are involved in upstream signal transduction of the PI3K/AKT/mTOR signal axis (Figure 3C).^[^ ^24^ ^]^

To test the hypothesis that the expression pattern of polarized genes in macrophages mediated by the chirality of self‐assembling matrixes may be related to adhesion and cytoskeletal remodeling,^[^ ^30^ ^]^ Subsequently, we focused on the expression of genes that related to adhesion and cytoskeletal remodeling. RT‐qPCR assays were performed to quantify the expression of mechano‐related genes in macrophages cultured with *P*‐type, *M*‐type, and *R*‐type self‐assembling matrixes, respectively. The expression of typical M2‐related genes and the mechanism pathway genes were significantly up‐regulated in the *M*‐type self‐assembling matrixes compared to the *P*‐type and *R*‐type self‐assembling matrixes (Figure 3F). Y27632 was used to antagonize the downstream signal of ROCK and BEZ235, as well as specifically block the downstream signal of PI3K and mTOR.^[^ ^25^ ^]^ There were no significant differences in the expression of M2‐related genes (ITGB1, CD44, RAC1, Vinculin, SOCS1, RhoA, mTOR, and IRF4) in *P*‐type, *R*‐type, and *M*‐type self‐assembling matrixes (Figure S24, Supporting Information), respectively. Moreover, the results that Y27632 inhibits M2 polarization of macrophages in chiral self‐assembling matrixes, indicate FAK/Rho/ROCK signal axe plays a key role in supramolecular chirality‐dependent macrophage polarization. Above them, our findings suggest that chirality‐dependent macrophage polarization is initiated by divergent mechanical sensations. The cell‐matrix interactions do not occur directly but *through* ligand‐integrin receptor binding.^[^ ^30^ ^]^ We characterized the affinity of matrix chirality to fibronectin (FN), the absorption assay showed significantly increased immunofluorescence staining of FN in the *M*‐type matrix, which indicated that the *M*‐type matrix facilitated an increased amount of ligand adsorption than the *P*‐type and *R*‐type matrixes (Figure S25, Supporting Information).

### 2.5 Classic Molecular Dynamic Simulation

**Figure 4:**
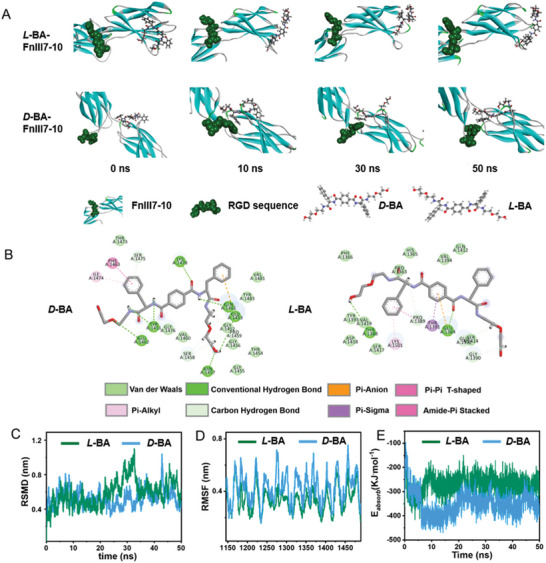
MD simulations proved the stereoselective interaction between FnIII7‐10 and *L*‐BA/*D*‐BA molecule. A) Snapshots. B) The binding structure in equilibrium between FnIII7‐10 and *L*‐BA/*D*‐BA molecule. C) The root‐mean‐square deviation (RMSD) D) and root‐mean‐square fluctuation (RMSF) values of the RGD residues in the FnIII7‐10 and *L*‐BA/*D*‐BA molecule. E) Computed average interaction energies between FnIII7‐10 and *L*‐BA/*D*‐BA molecule.

Classic molecular dynamic (MD) simulations were explored to gain more insight into the initial enantioselectivity between different chiral molecules (*L*‐BA/*D*‐BA) and FnIII7‐10 (as the key cell‐binding domains of fibronectin (FN), which plays a vital character in mediating cell‐material interaction^[^ ^7^, ^26^ ^]^). The snapshots demonstrated that the binding of *D*‐BA and FnIII7‐10 could reach a stable stage within 50 ns, while the interaction between *L*‐BA and FnIII7‐10 could not reach an equilibrium stage until 50 ns (**Figure** **4**A). This result suggested that FnIII7‐10 had a higher stereo‐affinity for *D*‐BA than *L*‐BA. The representative 3D and 2D structures of the adhesive domain complex indicate that greater coordination is achieved between *D*‐BA and FnIII7‐10 than between *L*‐BA and FNIII7‐10 (Figure 4B).^[^ ^7c^ ^]^ The smaller root‐mean‐square deviation (RMSD) value of FnIII7‐10 in the *D*‐BA/*L*‐BA and FnIII7‐10 complex (0.82 nm for the *L*‐BA and FnIII7‐10 complex *vs*. 0.65 nm for the *D*‐BA and FnIII7‐10 complex; Figure 4C) and the smaller root‐mean‐square fluctuation (RMSF) value of FnIII7‐10 in the *D*‐BA/*L*‐BA and FnIII7‐10 complex (0.60 for *D*‐BA and FnIII7‐10 complex *vs*. 0.42 nm for the *L*‐BA and FnIII7‐10 complex; Figure 4D) indicated that the high interdomain elasticity and flexibility of the RGD configuration in the *D*‐BA and FnIII7‐10 complex could facilitate integrins binding to the initial downstream cell response.^[^ ^7c^ ^]^ The lower interaction energy between FnIII7 and *D*‐BA (−105 kJ mol^−1^ for *L*‐BA and FnIII7‐10 complex, −181 kJ mol^−1^ for *D*‐BA and FnIII7‐10 complex, Figure 4E) further confirmed stronger binding of FnIII7‐10 on *D*‐BA than on *L*‐BA. Taken together, the MD simulation data confirmed a greater stereo‐affinity interaction of FnIII7‐10 to *D*‐BA than to *L*‐BA (Figure S26, Supporting Information), ultimately leading to the effects of chirality regulating the polarization of macrophage.

### 2.6 3D Chiral Self‐Assembling Matrixes on Enhance Repair of MI

**Figure 5:**
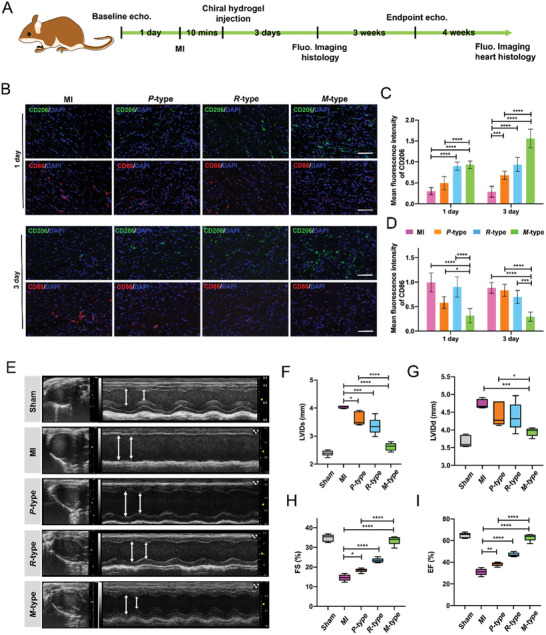
A) Time axis of the myocardial repair process detected in the MI region with implanting chiral matrixes. B) Immunofluorescence staining showed that the M1 macrophage‐related surface marker CD86 and M2 macrophage‐related surface marker CD206 were co‐stained in MI region after implantation of *M*‐type, *P*‐type and *R*‐type matrixes (scale :100 µm). C,D) Quantitative analysis showed the expression of CD206 and CD86 in *M*‐type, *P*‐type and *R*‐type matrixes. E) Echocardiography images of MI model of mice in each group 28 days after implanting chiral matrixes. F–I) Left ventricular internal diameter at the end‐systole (LVIDs), left ventricular internal diameter at the end‐diastole (LVIDd), left ventricular ejection fraction (LVEF), and left ventricular fraction shortening (LVFS) at 28 days after implanting chiral matrixes.

Macrophages are the primary responder cells of the immune system which infiltrate the infarcted myocardium for cardiac repair. After MI occurs, M1 macrophages mainly remove debris, necrotic cardiomyocytes (CMs), and apoptotic neutrophils, thereby causing inflammation. In the subsequent inflammation resolution stage, M2 macrophages dominantly promote tissue reconstruction and regeneration. During this process, the regulation of macrophage phenotype and inflammatory responses is critical for cardiac recovery.^[^ ^19^, ^27^ ^]^ Encourage by the promising in vitro results, we carried out the in vivo experiments employing the MI model of mice. A control group with PBS and self‐assembling matrixes groups were implanted into the MI region of mice, respectively, to study the regulation of macrophage phenotype and inflammatory responses in vivo (**Figure** **5**A). The proportion of CD206‐positive and CD86‐positive macrophages in the heart muscle was then assessed on days 1 and 3. Tissue immunofluorescence co‐staining and quantitative analysis showed that the proportion of CD206‐positive macrophages in *M*‐type self‐assembled matrices was significantly increased compared with *P*‐type and *R*‐type self‐assembled matrices (Figure 5B,C), while the proportion of CD86‐positive macrophages in *M*‐type self‐assembled matrices was significantly decreased compared with *P*‐type and *R*‐type self‐assembled matrices (Figure 5B,D). In addition, by evaluating the inflammatory factor iNOS and CD163 surface protein in the myocardial, the results showed that the proportion of CD163‐positive macrophages in the *M*‐type self‐assembly matrix was significantly increased compared with the *P*‐type and *R*‐type self‐assembly matrix, while the proportion of iNOS positive expression in the *M*‐type self‐assembly matrix was significantly decreased compared with the *P*‐type and *R*‐type self‐assembly matrix (Figure S27, Supporting Information). The comparison of biostabilities of *L*‐BA and *D*‐BA showed that the degradation rate of assemblies dramatically depended on their supramolecular chirality, namely, *M*‐type matrixes seemed to be more resistant to hydrolyzed than the *P*‐type matrix, which has been reported previously by our group.^[^ ^28^ ^]^ Additionally, quantitative IR783B‐NHS‐doped three self‐assembling matrixes were injected into the MI region of mice, respectively, and effective accumulation and degradation of the substances in the infarcted heart were observed (Figure S28, Supporting Information), which *M*‐type matrixes seemed to be more resistant to be hydrolyzed than the *P*‐type and *R*‐type matrixes. Moreover, the results of the detection of serum liver and kidney function in mice showed that the injection of chiral self‐assembling matrixes has no significant effect on the liver and kidney function of mice (Figure S29, Supporting Information). These data suggested the chiral self‐assembling matrixes have good biosafety. MI can cause severe pathological reactions, mainly ventricular remodeling thus changes in left ventricular (LV) thickness and mass.^[^ ^29^ ^]^ Subsequently, cardiac functions were evaluated 28 days after MI to verify the effect of chiral self‐assembling matrixes on the MI model of mice. The left ventricular function of different types of mice was analyzed by echocardiography (Figure 5E). Compared with the sham operation group without MI, the left ventricular diameter of all MI mice was increased, and the left ventricular systolic function was decreased. The *M*‐type self‐assembling matrixes showed the smallest increase in left ventricular internal diameter at the end‐diastole (LVIDd) and left ventricular internal diameter at the end‐systole (LVIDs) in all experiment groups. Compared with Sham, MI, *P*‐type, and *R*‐type self‐assembling matrixes, the infarcted hearts treated with the *M*‐type self‐assembling matrixes exhibited a significantly increased left ventricular ejection fraction (LVEF) and left ventricular fraction shortening (LVFS) (Figure 5F–I).

**Figure 6:**
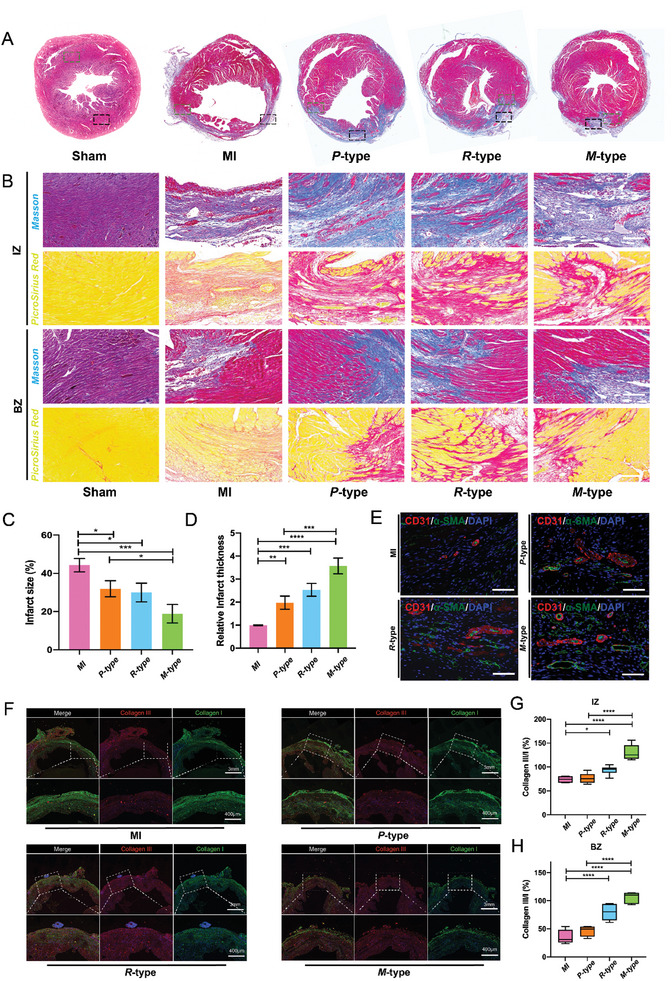
A) Panorama of Masson's trichromatic horizontal heart images at 28 days postoperatively. B) Masson trichromatic and Sirius red staining were used to evaluate the anti‐fibrosis effect of chiral matrixes, Scale bar: 50 µm. C) Percentage of the fibrotic area in each left ventricle after coronary artery ligation. D) Quantitative analysis of myocardial remodeling by ventricular wall thickness in infarct area. E) α‐SMA and CD31 immunofluorescence co‐staining were performed 28 days after implanting chiral matrixes, Scale bar: 50 µm. F). On day 28, the distribution of type III collagen and type I collagen levels in IZ region in MI, *M*‐type, *P*‐type and *R*‐type matrixes groups. G‐H) Quantitative analysis of the ratio of type III collagen to type I collagen in IZ and BZ region was measured in the MI, *M*‐type, *P*‐type and *R*‐type matrixes groups, Scale bar: 3 mm and 400 µm.

In the pathologic process of MI, excessive degradation of ECM adversely affects cardiac function.^[^ ^30^ ^]^ Herein, 3D chiral self‐assembling matrixes provide structural support for the infarcted tissue microenvironment and further, promote the repairing of the MI region. Masson is trichromatic and Sirius red staining was used to analyze the cardiac fibrosis after 28 days of treatment (**Figure** **6**A). Compared with the *MI* group, the injection of chiral self‐assembling matrixes groups inhibited the thinning of the left ventricular (LV) wall, as well as the *M*‐type self‐assembling matrixes has the most significant inhibitory effects (Figure 6B). Quantitative analysis correspondingly showed the *M*‐type self‐assembling matrixes had the least thinning and fibrosis of the LV wall compared with *MI*, *R*‐type, and *P*‐type matrixes (Figure 6C,D). The regeneration of blood vessels is crucial in the process of repairing MI. Finally, an immunohistochemical examination was performed 4 weeks later, and tissue sections of the site of MI were taken to evaluate the degree of fibrosis, which CD31 and α‐SMA could as the index of cardiac regeneration. The expression of CD31 and α‐SMA was significantly higher in the *M*‐type self‐assembling matrixes than in the *MI* group, *P*‐type, and *R*‐type self‐assembling matrixes (Figure 6E). These results were consistent with the above echocardiographic data. Pathological diagnosis of hematoxylin‐eosin (HE) staining showed there was no significant toxicity to the heart of injected mice with different chiral self‐assembling matrixes treatments compared to control groups (Figure S30, Supporting Information). In addition, collagen is mainly distributed in IZ and BZ. On day 28, the levels of *P*‐type, *R*‐type, and *M*‐type group type III collagen and type I collagen were higher than those in the *MI* group. We further detected collagen subtypes by immunostaining. The ratio of type III/type I collagen in *M*‐type was significantly higher than in the other groups (Figure 6F), IZ (Figure 6G), and BZ (Figure 6H). The implanted bionic 3D chiral self‐assembling matrixes developed in this work could achieve improved cardiac function without the assistance of stem cells, drugs, or genes. The significantly improved restoration of cardiac function further confirms the advantages and application prospects of chiral self‐assembling matrixes in cardiac repair.

## 3 Conclusion

In summary, bionic 3D ECM based on chiral supramolecular matrixes is employed to manipulate the regulation of macrophages’ immune response as well as enhance the repair of myocardial infarction. Results imply that *M*‐type self‐assembling matrixes significantly inhibit inflammation and promote damaged myocardial repair by upregulating M2 macrophage polarization and downstream immune signaling compared with *P*‐type and *R*‐type self‐assembling matrixes. The corresponding infarct size was reduced, and the thickness of the left ventricular wall was increased substantially in the MI model with implanting chiral self‐assembling matrixes. The significantly improved recovery of cardiac function further verified the excellent therapeutic efficacy of *M*‐type matrixes in cardiac repair. MD data demonstrate that *M*‐type self‐assembling matrixes display higher stereo‐affinity to cellular binding compared to *P*‐type and *R*‐type self‐assembling matrixes, which enhance the clustering of mechanosensitive Itgβ1, activate FAK and Rho‐ROCK signaling pathway, as well as downstream PI3K/AKT/mTOR signaling axes to promote M2 polarization. This work revealed that the 3D supramolecular chirality microenvironment of biomaterials could serve as a promising regulator to affect macrophage behavioral responses and provide insights for the design of immunoregulatory biomaterials for the intended use.

## Conflict of Interest

The authors declare no conflict of interest.

## Supporting information

Supporting Information

## Data Availability

Research data are not shared.
