# Supplementary material for: 3D Chiral Self‐Assembling Matrixes for Regulating Polarization of Macrophages and Enhance Repair of Myocardial Infarction
Source: Adv Sci (Weinh). 2023 Sep 28;10(32):2304627. doi: 10.1002/advs.202304627 (PMC10646248; doi:10.1002/advs.202304627)
Supplement: Supplementary file 1 — Supporting Information [file ADVS-10-2304627-s001.pdf]

ADVANCED SCIENCE
Open Access

Supporting Information

for *Adv. Sci.*, DOI 10.1002/advs.202304627

3D Chiral Self-Assembling Matrixes for Regulating Polarization of Macrophages and Enhance Repair of Myocardial Infarction

Lei Yang, Li Yang, Kongli Lu, Nan Su, Xueqin Li, Shuoxiang Guo, Song Xue, Feng Lian* and Chuanliang Feng**

Supporting Information

Three-Dimensional Chiral Self-Assembling Matrixes for Regulating Polarization of Macrophages and Enhance Repair of Myocardial Infarction

Lei Yang, Li Yang, Kongli Lu, Nan Su, Xueqin Li, Shuoxiang Guo, Song Xue, Feng Lian*, and Chuanliang Feng**

Materials

Starting materials were purchased from commercial suppliers and used without further purification unless otherwise stated. Tert-butyloxycarbonyl-*L*-phenylalanine (Boc-*L*-PhOH), diglycolamine, triethylamine (Et_3N), trifluoroacetic acid (TFA), 1,4- benzenedicarbonyl dichloride, 1-hydroxybenzotriazole (HOBt), citric acid, 3-(3-dimethyl aminopropyl)-1-ethyl carbodiimide hydrochloride (EDCI), sodium bicarbonate ($NaHCO_3$), sodium chloride (NaCl), sodium sulfate (Na_2SO_4), ethyl acetate, petroleum ether, dichloromethane (DCM), and dimethyl sulfoxide (DMSO) were purchased from Adamas-Beta Reagent Co. Ltd. (Shanghai, China). Lipopolysaccharide (LPS from Salmonella enterica serotype enteritidis), 4% isofurane were purchased from Gibco by Life Technologies. EZ-Press RNA Purification Kit (EZBioscience, Fairview Ave, USA), PrimeScrip RT-PCR Kit (TaKaRa, Shiga, Japan), SYBR premixed Ex Taq (TaKaRa, Shiga, Japan), DMEM (Invitrogen), FBS (Invitrogen), penicillin, streptomycin, Cell Counting Kit-8 assay kit, live/dead kit reagents (Invitrogen), CD163 (1:200; Cambridge, UK, Abcam), iNOS (1:200; Cambridge, UK, Abcam), (Alexa Fluor 647 - Goat Anti-Rabbit IgG, Cambridge, UK, Abcam, 1:800), DAPI dye (Invitrogen), Phalloidin (Invitrogen), BCA Protein Assay Kit (Thermo Fisher Scientific, Rockford, IL, USA), PI3K (1: 1000; Cambridge, UK, Abcam), P-PI3K (1: 1000; Cambridge, UK, Abcam), FAK (1: 1000; Cambridge, UK, Abcam), P-FAK (1: 1000; Cambridge, UK, Abcam), AKT (1: 1000; Cambridge, UK, Abcam), P-AKT (1: 1000; Cambridge, UK, Abcam) and GAPDH (1: 500; Cambridge, UK, Abcam). Goat anti-Rabbit IgG (H + L) Highly Cross-Adsorbed Secondary Antibody Alexa Fluor 488, F4/80 (1:200; Cambridge, UK, Abcam), CD206 (1:200; Cambridge, UK, Abcam), CD68 (1:200; Cambridge, UK, Abcam) and iNOS (1:200; Cambridge, UK, Abcam), CD31 (1:200; Cambridge, UK, Abcam), α-SMA (1:200; Cambridge, UK, Abcam).

Methods

Circular Dichroism (CD) spectroscopy: The CD spectra of self-assembled hydrogels were obtained using JASCO J-1500 CD spectrometer (Japan) and analyzed with the supplied Spectra-Manager software. The spectra were recorded from a wavelength 600 to 200 nm with bandwidth of 0.5 nm.

Rheological measurements: Rheological measurements were performed using a rotary rheometer (Kinexus rotational rheometer from Malvern) with steel plate-plate geometry (diameter: 25 mm). Hot solution of all samples was poured onto the plate of the instrument and covered by a solvent trap for about 30 min to ensure homogeneous temperature and the formation of stable self-assembled hydrogels. All measurements were conducted with a 1.0 mm gap distance between the plates. All experiments were carried out within the linear viscoelastic regime (LVR), in which the measured shear moduli (G', G'') are independent of the applied strain (i.e. without disruption of the gel structure). Strain sweeps were conducted from 0.01%-100% at a frequency of 6.28 rad s^{-1}. Frequency sweeps were performed at a constant strain of 0.5% and frequencies between 0.1 and 100 rad s^{-1}.

Calculation of the value of g_{abs}: The g_{abs} value is defined as Δε divided by ε. It is generally expressed by the following equation: gabs = Δε/ε = ΔAbs/Abs. The gabs value represents the degree of asymmetry of the absorption band. Experimentally, the equation for calculating the gabs value in each data mode is given below: g_{abs} = CD(mdeg)/(32980*Abs)=ΔOD/Abs=Spc.Ellip/(32980*Spec.Abs) = Mol.CD/Mol.Abs = Mol.Ellip/(3298*Mol.Abs). Actually, the gabs can be directly read by a JASCO CD spectrophotometer. For a CD spectrophotometer (like Chirascan, Applied Photophysics Ltd., UK) could only provide CD (unit: mdeg) and Abs value, the equation of gabs is applied, which has also been reported by other groups. [3-4]

3D macrophages culture in chiral supramolecular hydrogels

Raw 264.7 macrophage culture: Raw 264.7 murine mononuclear macrophages were purchased from the Chinese Academy of Sciences and cultured in DMEM (pH 7.4) containing 10% FBS, 100 units /mL penicillin and 100 μg/mL streptomycin in a controlled incubator at 37 °C with atmospheric carbon dioxide content of 5%.

Live-dead assay and Cell Counting Kit 8 assay the live-dead assay was carried out as described above. Each well (1×10^5 cells per well) was cultured with P-type, R-type and M-type (50 μL) thin films on 24-well plates. Next, each well was incubated with live/dead kit reagents (Invitrogen) according to the recommended measurements. Images were obtained after 10 min of fluorescence microscopy staining. Cell Counting Kit 8 (CCK-8) detects cell proliferation. Under proliferation condition, macrophages, cardiomyocytes and cardiac fibroblasts were inoculated into 96-well plates at the rate of 1×10^4 cells/well. After incubation for 0 h, 24 h, 48 h, and 72 h, the CCK-8 reagent was incubated in culture for 4 h, and the optical density was measured at 450 nm using a microplate meter (ELX800, Bio Tek, USA).

Total RNA Separation and quantitative polymerase chain reaction (qPCR) Total RNA was extracted from macrophages using standard methods and PCR using primers. The RNA was collected and purified using the EZ-Press RNA Purification Kit (EZBioscience, Fairview Ave, USA) according to the manufacturer's instructions. The total RNA concentration was quantified using a NanoDrop spectrophotometer. RNA was reverse-transcribed into cDNA using PrimeScrip RT-PCR Kit (TaKaRa, Shiga, Japan). The quantitative real-time PCR reaction was performed using a Light Cycler from Roche Molecular Biochemicals, using SYBR premixed Ex Taq (TaKaRa, Shiga, Japan), according to the protocol provided by the manufacturer. The PCR reaction process was as follows: denaturation at 95 °C for 10 min (initial denaturation) followed by 30 cycles, denaturation at 95 °C for 30 s, then annealing at 57 °C for 30 s, and elongation at 72 °C for 30 s. GAPDH is used as an internal standard. A no template blank and a reverse transcriptional negative blank served as a negative control. 2∧(-ΔΔCt) method was used to process the data. Gene expression of each factor was normalized by GAPDH. All the measurements were made in triplicates. The sequence of target primers is shown in Table S2.

ELISA: All groups were treated with the previous culture method. After 48 hours of cell culture, the supernatant of cell culture medium was collected for ELISA. Then, 150 μL standard solutions of IL-1β, IL-6, IL-12 and TNF-α (480 ng/mL) were added into Eppendorf test tube as reference standard. 150 μL of standard diluent was added to the solution and rotated for 30 s to obtain 240 ng/mL standard stock solution. Then, the stock solutions of 120-ng/mL, 60-ng/mL, 30-ng/mL and 15-ng/mL were prepared in Eppendorf test tube by continuous dilution with 150 μL of reference standard dilution. Finally, according to the manufacturer's instructions, the

concentrations of IL-1β, IL-6, IL-12 and TNF-α in the supernatant of cell culture medium were determined by ELISA.

Immunofluorescence cell suspension (1× 10^5 cells per well) was inoculated on the coated 24-well plate and cultured for 72 h. Immunofluorescence was used to detect the ability of chiral self-assembling matrixes to promote the polarization of macrophages towards M2. The cells were fixed with 4% paraformaldehyde for 30 min, then treated with 0.03% Triton X-100 phosphate buffer and sealed with 10% normal goat serum for 1 h. Next, the samples were incubated overnight with primary antibodies (1:200 dilution), such as anti-CD163 (1:200; Cambridge, UK, Abcam) and anti-iNOS (1:200; Cambridge, UK, Abcam), at 4 °C. The cells were then incubated in the dark for 1 hour with a secondary antibody (Alexa Fluor 647 - Goat Anti-Rabbit IgG, Cambridge, UK, Abcam, 1:800). The nuclei were stained with DAPI dye (Invitrogen), the cytoskeleton was stained with Phalloidin (Invitrogen), and the stained cells were observed by fluorescence microscope. Finally, Image J software is used to analyze the data.

Gene expression analysis: Macrophages (2 × 10^5 cells per well) were inoculated on 24-well plates and differentiated for 3 days' culture. Total RNA was extracted by extraction kit method. Detection methods: Nanodrop2000 was used to detect the concentration of the extracted nucleic acid, Agient2100 was used to detect the integrity of Lab Chip GX, and gel electrophoresis was used to detect impurity contamination. Damage Repair, End Repair and joint connection of mixed products are carried out for qualified samples using NEB Next FFPE DNA Repair Mix and NEB Next Ultra II End Repair Module. Sqk-lsk109 (ONT) kit was used to bind the library before loading. The final reaction products were placed on the PromethION48 sequencer for on-machine sequencing (Beijing BIOMARKER Biotechnology Co., LTD.).

Western Blotting the cell suspension (1× 10^5 cells per well) was inoculated on the coated 24-well plate and cultured for 72 h. With RIPA lysis buffer containing protease inhibitor for 15 min, followed by ultrasonic treatment for 5 min, centrifuged at 12,000 g for 15 min, and the supernatant collected. The protein concentration was determined using the Pierce BCA Protein Assay Kit (Thermo Fisher Scientific, Rockford, IL, USA), and then 5×SDS-PAGE non-reducing protein loading buffer was added and boiled at 95 °C for 10 min. Equivalent protein samples were separated by 10% sodium dodecyl sulfate-polyacrylamide gel electrophoresis (SDS-PAGE) and transferred to polyvinylidene fluoride (PVDF) membrane. The membranes

were blocked using 5% BSA and incubated with primary antibodies against PI3K (1: 1000; Cambridge, UK, Abcam), P-PI3K (1: 1000; Cambridge, UK, Abcam), FAK (1: 1000; Cambridge, UK, Abcam), P-FAK (1: 1000; Cambridge, UK, Abcam), AKT (1: 1000; Cambridge, UK, Abcam), P-AKT (1: 1000; Cambridge, UK, Abcam) and GAPDH (1: 500; Cambridge, UK, Abcam). Then the membrane was incubated with Goat anti-Rabbit IgG (H + L) Highly Cross - Adsorbed Secondary Antibody, Alexa Fluor 488 second incubation for 1h at room temperature, using the LI-COR company's Odyssey two-color infrared fluorescence imaging system scans.

In vivo experiment section: In vivo toxicity Blood analysis (hematological and serum chemical analysis): Twenty-four healthy 7-week-old female C57 mice were selected for studying the toxicological of chiral self-assembling matrixes. PBS without chiral self-assembling matrixes was injected into the hearts of 6 mice, and 20 μL P-type, R-type and M-type chiral self-assembling matrixes was injected into the hearts of the other mice, respectively. After 3 days, half of the mice were collected from each group, and blood was collected from each mouse (3 mice in each group, at the same time). The blood was placed at room temperature for 2 h, followed by centrifugation of 1000 rpm at 4°C for 15 min. Then the supernatant was analyzed by serum chemistry. After 28 days, the other half of the mice were euthanized. After the heart was removed, HE is staining was performed to analyze the injection area.
Assessment of cardiac function at 28 days after myocardial infarction, the mice were examined by m-mode and 2-D transthoracic echocardiography. Briefly, echocardiography was performed in mice under 2-3% isoflurane anesthesia. An VEVO LAZR-X (VEVO LazR-X multi-mode ultrasound/photoacoustic imaging system, Fujifilm VisualSonics, USA) was used to detect cardiac function.

Histological Analysis After echocardiography, the mice were euthanized and their hearts were removed and fixed with 4% paraformaldehyde. Then, the heart was sectioned from the apex to the ligation site (5μm) and embedded in paraffin. Sirius red and Masson trichromatic staining were used to analyze wall thickness and collagen deposition. The ratio of infarcted left ventricular wall thickness to normal left ventricular wall thickness was calculated. Masson trichromatic staining was used to analyze collagen deposition and calculate the ratio of collagen deposition to the proportion of the whole left ventricle. The wall thickness and collagen content were calculated by ImageJ. To evaluate the polarization effect of chiral self-assembling matrixes on cardiac macrophages, we selected mice injected for 3 days and analyzed the left

ventricular tissue sections with F4/80 (1:200; Cambridge, UK, Abcam), CD206 (1:200; Cambridge, UK, Abcam), CD68 (1:200; Cambridge, UK, Abcam) and iNOS (1:200; Cambridge, UK, Abcam) antibodies. To evaluate the cardiovascularization effect of chiral nanofibers, left ventricular tissue sections were analyzed using platelet endothelial cell adhesion molecule-1 (PECAM-1/CD31) (1:200; Cambridge, UK, Abcam) and α-smooth muscle actin (α-SMA) (1:200; Cambridge, UK, Abcam) antibodies. Images of blood vessels in 5 regions were obtained randomly, and the number of blood vessels was expressed as area %.

Statistical analysis All data were expressed as mean ± SEM of three independent experiments. The n value represents the number of independent experiments conducted, the number of individual experiments, or the number of mice. $P < 0.05$ was considered significant (*$p < 0.05$, **$p < 0.01$, ***$p < 0.001$, ****$p < 0.0001$). One-way analysis of variance (ANOVA) was used for the analysis of significant differences between groups. After confirming the normal distribution, Tukey multiple comparison test and Student's t-test were used for the double-component comparison. Prism GraphPad Software was used for statistical analysis.

Myocardial Infarction Model and Treatment: Male C57/L mice were used to prepare the MI animal model. The mice were anesthetized by intraperitoneal injection of 2% sodium pentobarbital. The heart was exposed from the left thoracotomy and the proximal left anterior descending artery (LAD) was ligated with 6-0 polypropylene. The success of MI was confirmed by the typical ST segment elevation in electrocardiography. The MI model mice were randomly divided into 4 groups (n = 10 each group) as follows, (1) sham (no MI, underwent thoracotomy only without LAD ligation); (2) PBS (100 μL); (3) Alg hydrogel (100 μL); (4) MNPs/Alg hydrogel (100 μL). The hydrogels were rapidly injected in the border of the infarct area at three different locations with a 28-gauge needle. Then, chest was closed in multiple layers. Mice were sacrificed on Days 1, 3, and 28, respectively after different treatments. Heart tissues were obtained for analysis. All animal procedures were performed in accordance with NIH guidelines (Guide for the care and use of laboratory animals) and approved by Institutional Animal Care and Use Committee of Shanghai Jiao Tong University School of Medicine (License: SYXK (Shanghai) 2016–0009, Ethic code: A2020124).

Synthesis and characterization of *D*/*L*-BA hydrogelators.

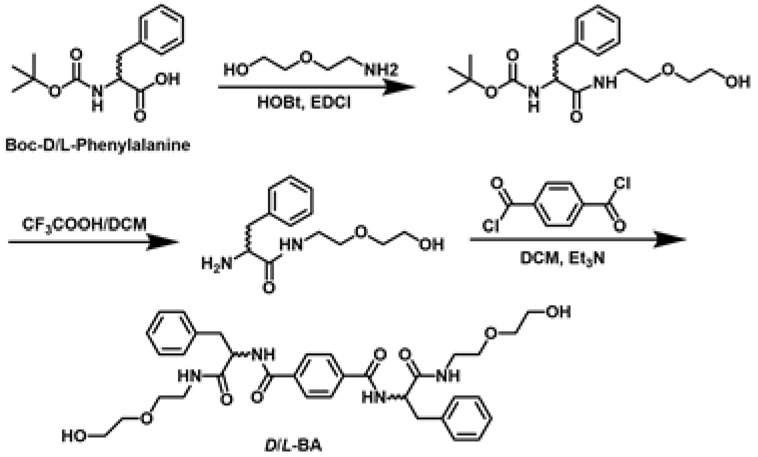

Scheme S1. Synthesis procedures of *D/L*-BA.

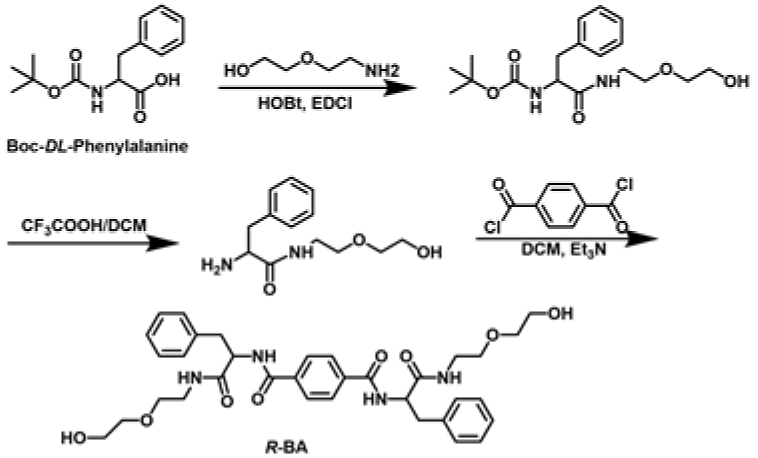

Scheme S2. Synthesis procedures of *R*-BA.

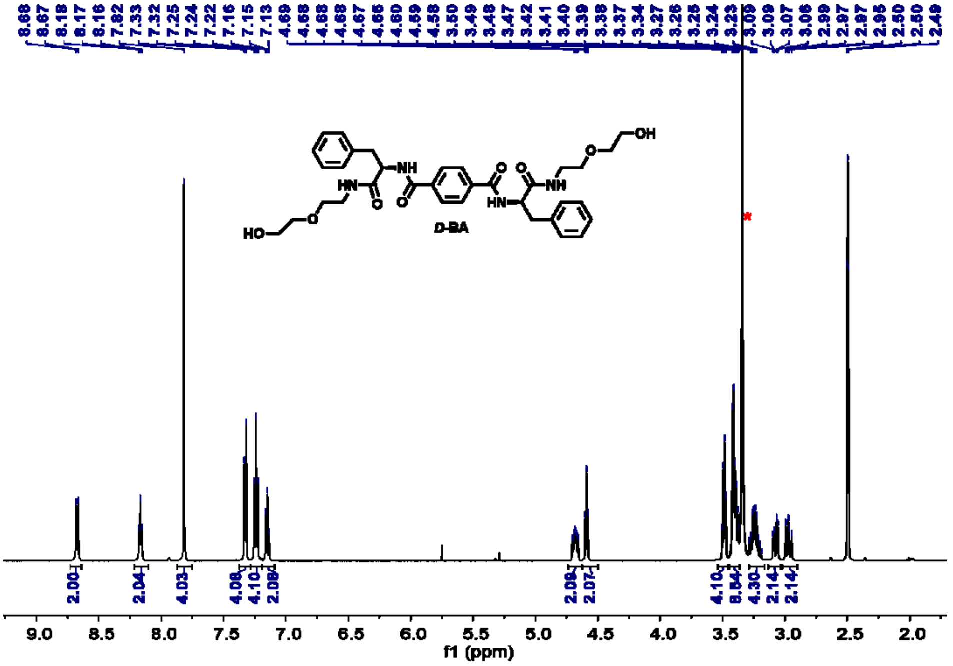

Figure S1. ^{1}H-NMR spectrum of *D*-BA (500 MHz, DMSO-d$_6$). δ 8.67 (d, J = 8.5 Hz, 2H), 8.17 (t, J = 5.5 Hz, 2H), 7.82 (s, 4H), 7.33 (d, J = 7.0 Hz,4H),7.24 (t, J = 7.5 Hz, 4H), 7.15 (t, J = 7.5 Hz, 2H), 4.69 (ddd, J = 9.5, 4.5, 2Hz, 2H), 4.59 (t, J = 5.5 Hz, 2H), 3.50 (dd, J = 7.7 Hz, 4H), 3.42-3.34 (m, 8H), 3.26 (m, 4H), 3.07 (dd, J = 9.5, 4.5 Hz, 2H), 2.97 (t, J = 12 Hz, 2H).

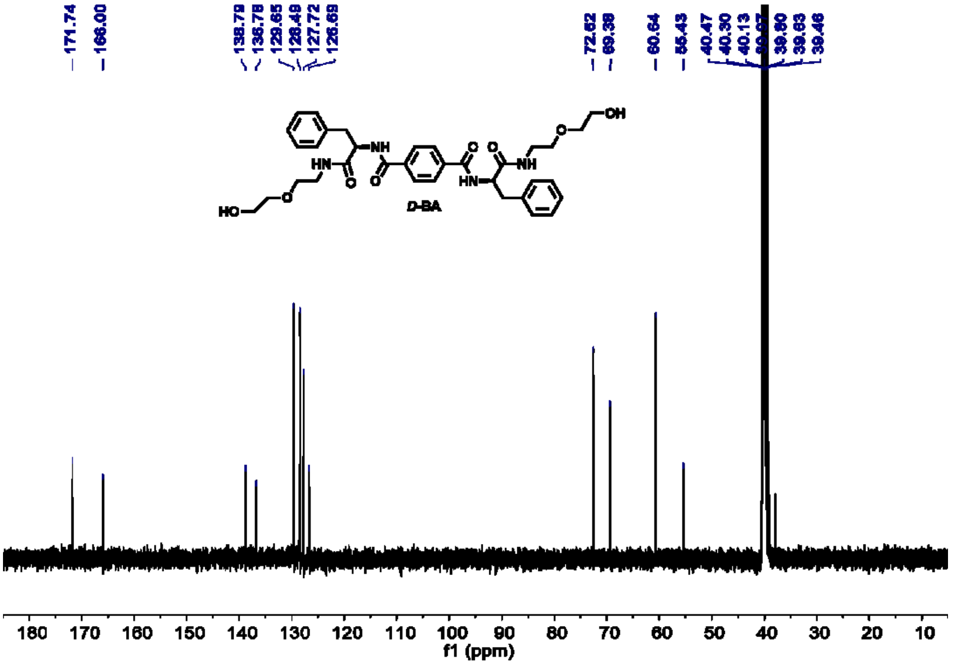

Figure S2. ^{13}C-NMR spectrum of *D*-BA (125 MHz, DMSO-d6). δ 171.45, 165.68, 138.48, 136.45, 129.33, 128.17, 127.41, 126.36, 72.30, 69.06, 60.32, 55.12, 38.85, 37.54.

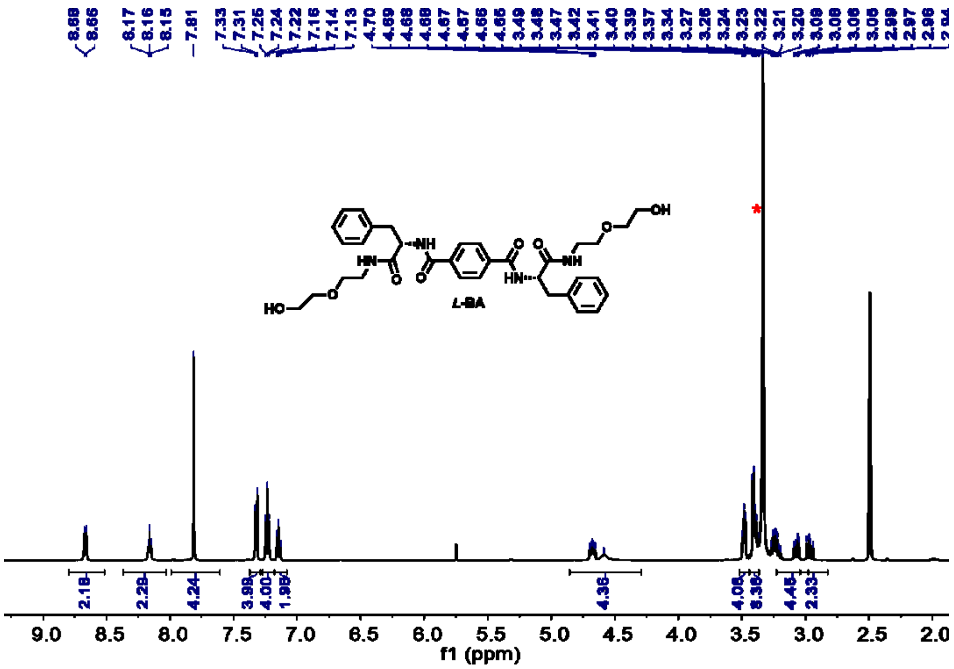

Figure S3. ^{1}H-NMR spectrum of *L*-BA in DMSO-d$_6$. ^{1}H NMR (500 MHz, DMSO-d$_6$) δ 8.67 (d, J = 8.5 Hz, 2H), 8.16 (t, J = 5.5 Hz, 2H), 7.81 (s, 4H), 7.32 (d, J = 7.0 Hz,4H),7.24 (t, J = 7.5 Hz, 4H), 7.15 (t, J = 7.5 Hz, 2H), 4.69 (ddd, J = 9.5, 4.5, 2Hz, 2H), 4.58 (t, J = 5.5 Hz, 2H), 3.43-3.36 (m, 8H), 3.26 (m, 4H), 3.08 (dd, J = 9.5, 4.5 Hz, 2H), 2.98 (t, J = 12 Hz, 2H).

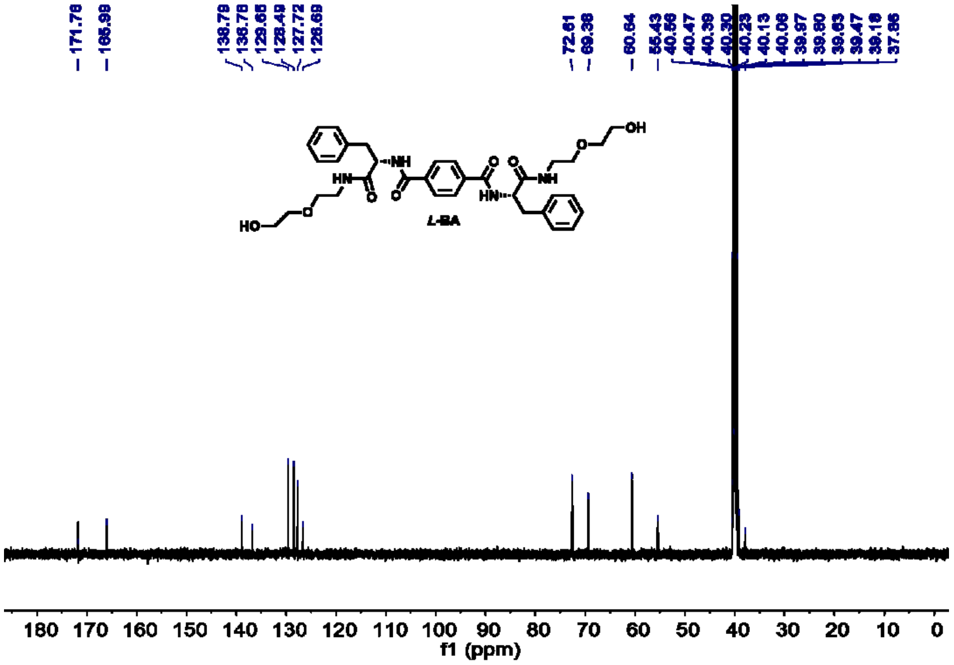

Figure S4. ^{13}C-NMR spectrum of *L*-BA in DMSO-d$_6$. ^{13}C NMR (125 MHz, DMSO-d6) δ 171.45, 165.68, 138.48, 136.45, 129.33, 128.17, 127.41, 126.36, 72.30, 69.06, 60.32, 55.12, 38.85, 37.54.

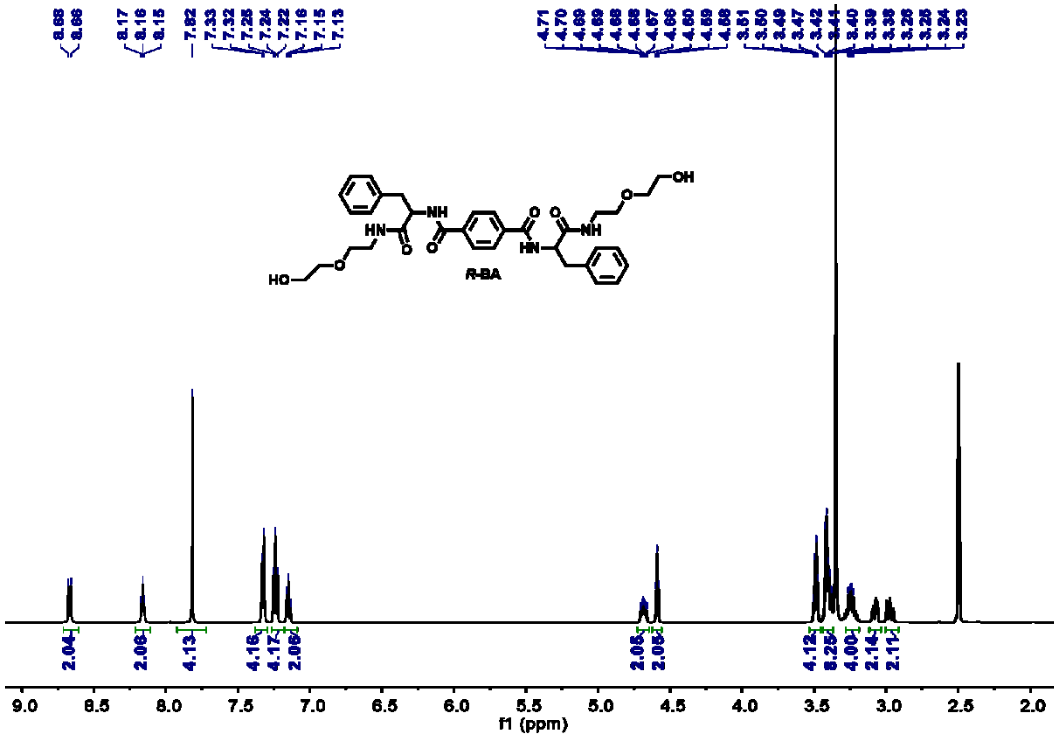

Figure S5. ^{1}H-NMR spectrum of *R*-BA in DMSO-d$_6$ (500 MHz, DMSO-d$_6$) δ 8.67 (d, J = 8.5 Hz, 2H), 8.17 (t, J = 5.5 Hz, 2H), 7.82 (s, 4H), 7.33 (d, J = 7.0 Hz,4H),7.24 (t, J = 7.5 Hz, 4H), 7.15 (t, J = 7.5 Hz, 2H), 4.69 (ddd, J = 9.5, 4.5, 2Hz, 2H), 4.58 (t, J = 5.5 Hz, 2H), 3.43-3.36 (m, 8H), 3.26 (m, 4H), 3.08 (dd, J = 9.5, 4.5 Hz, 2H), 2.98 (t, J = 12 Hz, 2H).

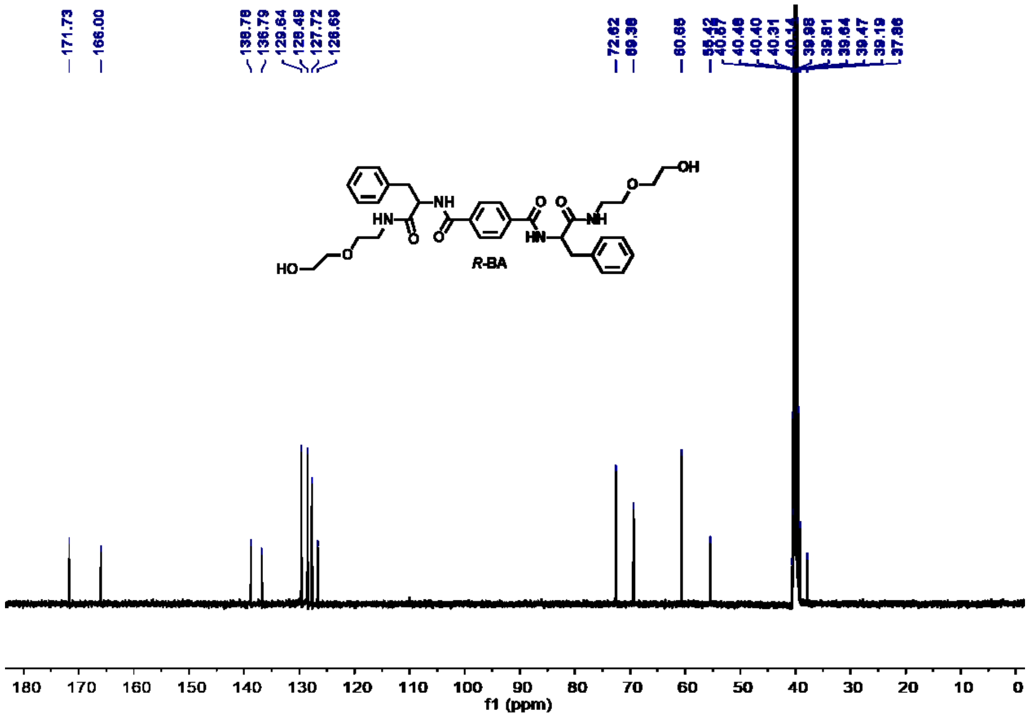

Figure S6. ^{13}C-NMR spectrum of *R*-BA in DMSO-d_6. ^{13}C NMR (125 MHz, DMSO-d6) δ 171.45, 165.68, 138.48, 136.45, 129.33, 128.17, 127.41, 126.36, 72.30, 69.06, 60.32, 55.12, 38.85, 37.54.

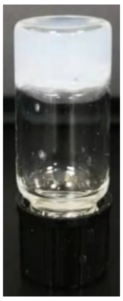

Figure S7. Photographic images of the free-standing *M*-type self-assembling matrixes of *D*-BA in PBS (pH=7.4) at room temperature, respectively.

Table S1. Gelation ability of *D*-BA, *L*-BA, and *R*-BA in water, respectively (pH =7.4).

Entry	Gelator	[a] Concentration (mg.mL^{-1})	Condition	Time	Stability
1	*D*-BA	0.3	Solution	-	-
2	*D*-BA	1.0	Full Gels	10 min	> 6 months
3	*D*-BA	3.0	Full Gels	2 min	> 6 months
4	*D*-BA	4.0	Gels +P	-	-
4	*L*-BA	0.3	Solution	-	-
5	*L*-BA	1.0	Full Gels	10 min	> 6 months
6	*L*-BA	3.0	Full Gels	2 min	> 6 months
7	*L*-BA	4.0	Gels +P	-	-
8	*R*-BA	0.3	Solution	-	-
9	*R*-BA	1.0	Full Gels	10 min	> 6 months
4	*R*-BA	3.0	Full Gels	2 min	> 6 months
5	*R*-BA	4.0	Gels +P	-	-

[a] 3% DMSO in the PBS, P: precipitate

Supplementary Figures

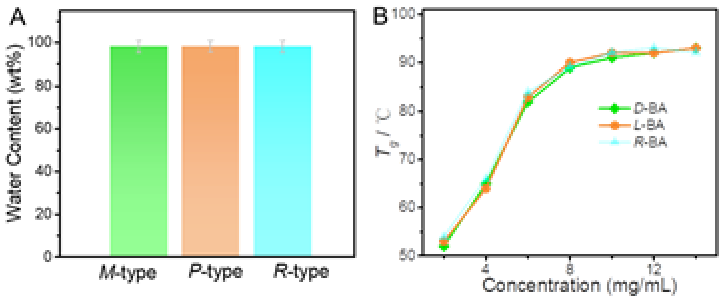

Figure S8. The water content and denaturation temperature of *L*-BA, *D*-BA, and R-BA gels, respectively.

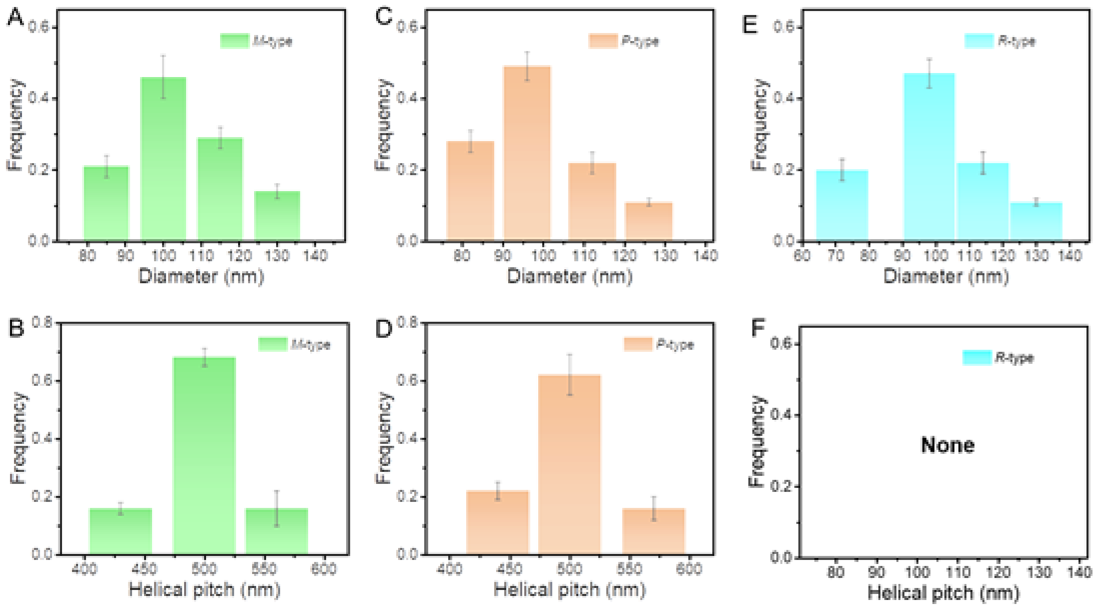

Figure S9. Corresponding diameter and helical pitch distributions of nanostructures obtained from *M*-type, *P*-type, and *R*-type hydrogels, respectively.

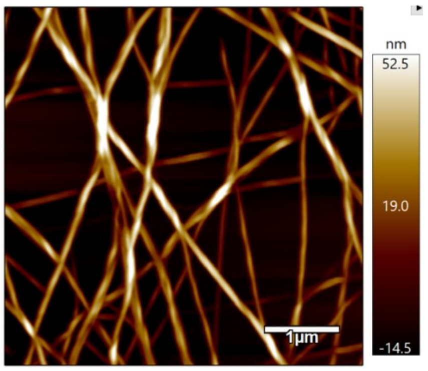

Figure S10. AFM image of *R*-type matrixes obtained from the self-assembly of *R*-BA.

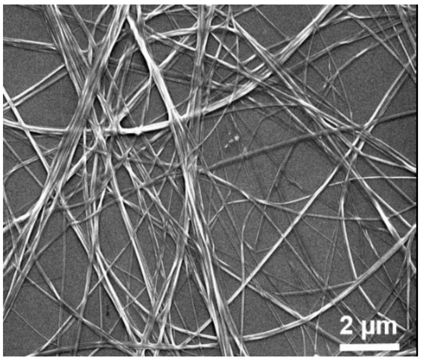

Figure S11. SEM images of *R*-type matrixes obtained from the self-assembly of *R*-BA.

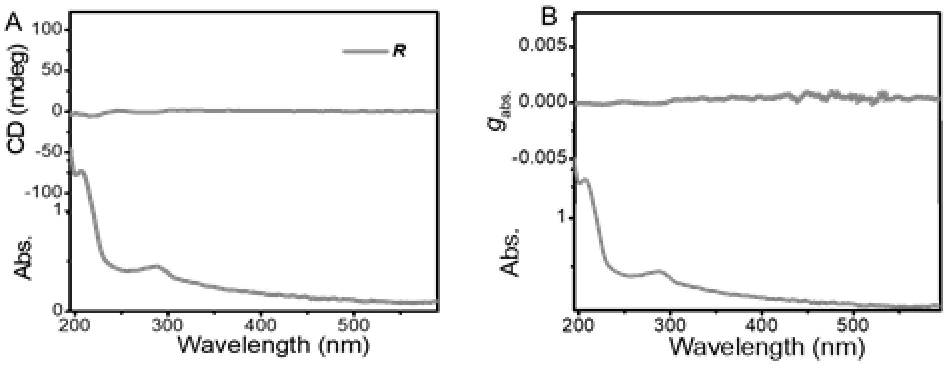

Figure S12. Corresponding CD and g_{abs} spectroscopy of *R*-type self-assembling matrixes.

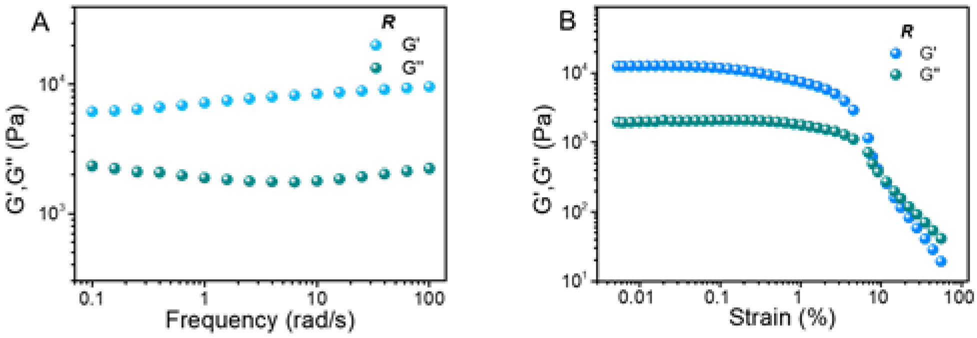

Figure S13. The mechanical properties of *R*-type self-assembling hydrogels was measured by a rotary rheometer with dynamic frequency sweep at a strain of 0.01%-100%.

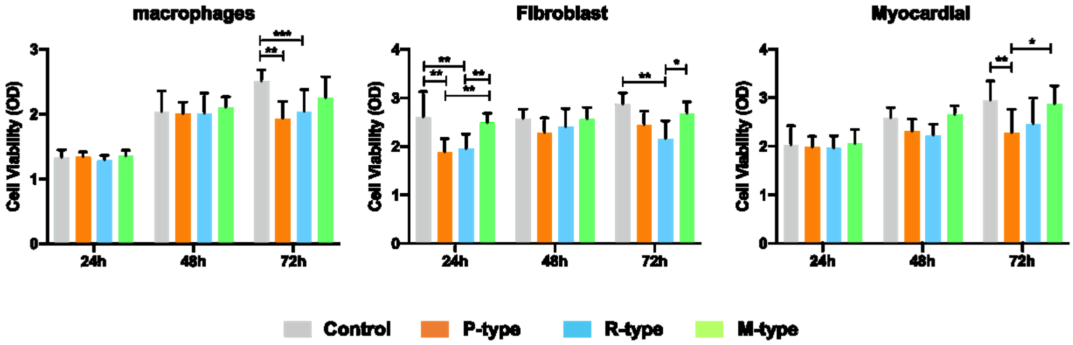

Figure S14. CCK-8 experiment showed that the activities of macrophages, fibroblasts and cardiomyocytes were cultured in different chiral matrixes environments.

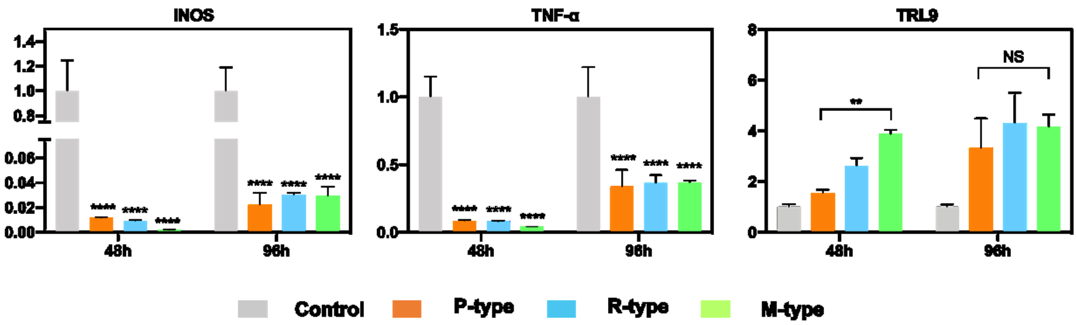

Figure S15. RT-qPCR analysis showed the inflammatory genes iNOS and TNF-α in *M*-type matrixes were significantly lower than those in *P*-type and *R*-type matrixes, as well as M2-related gene TLR9 was significantly increased.

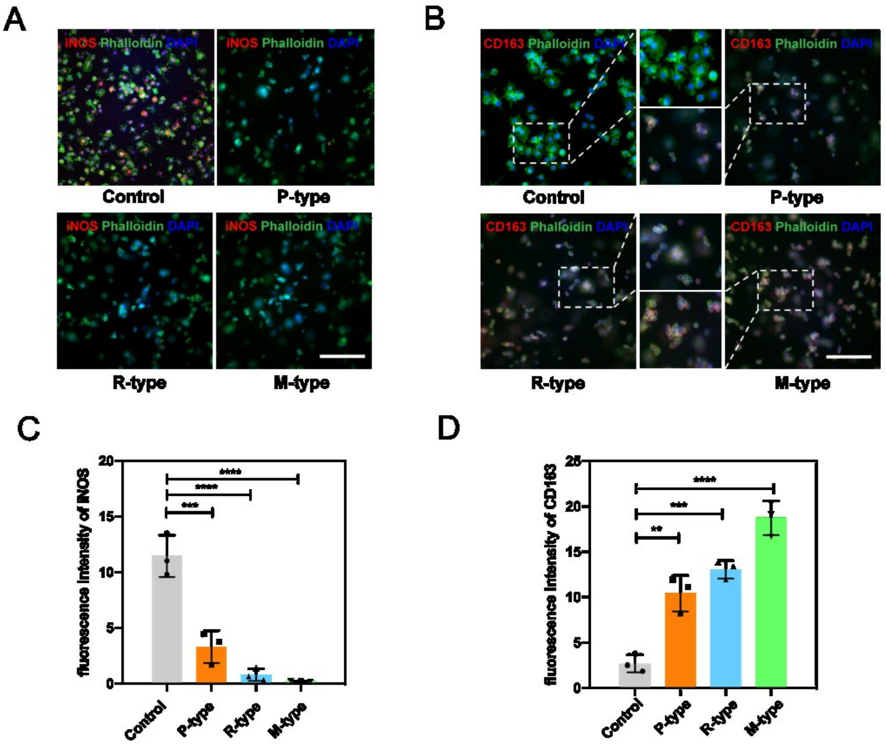

Figure S16. Immunofluorescence was used to analyze the changes in cell phenotype and the expression of membrane protein after the polarization of macrophages. scale :20 μm

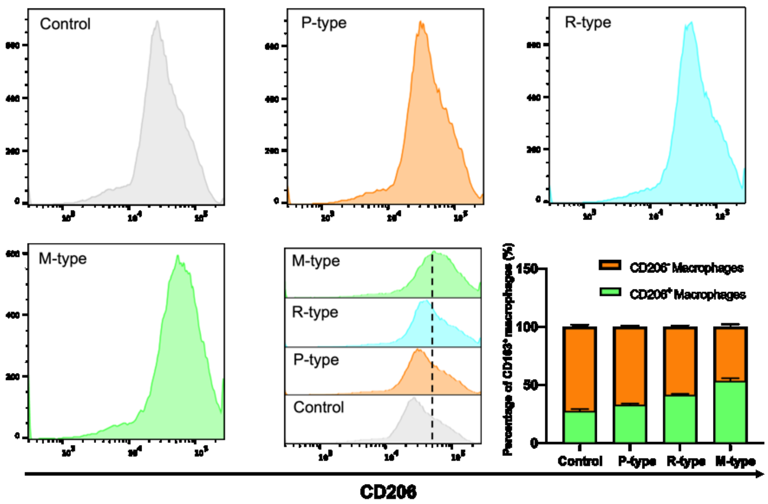

Figure S17. Flow cytometry also confirmed that *M*-type matrixes promoted the expression of CD206 more easily than *R*-Type and *P*-type matrixes.

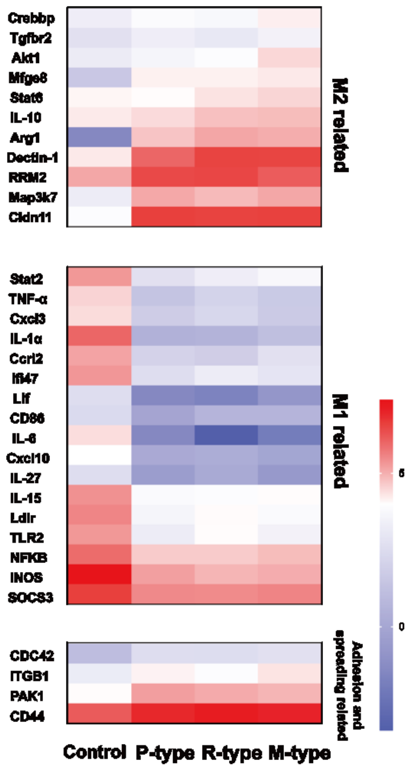

Figure S18. Hierarchical clustering showed M2-related genes were up-regulated while M1-related genes were down-regulated in P-type, M-type, and R-type matrixes, respectively, among them, the changes in M-type matrixes were the most significant

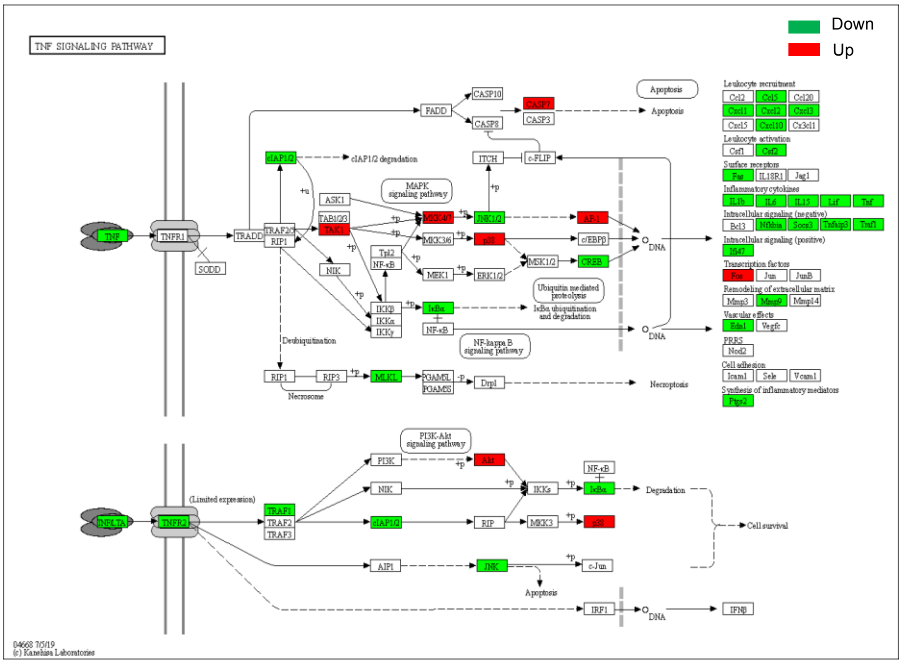

Figure S19. Gene ontology analysis and pathway enrichment analysis showed that TNF signaling pathway were activated.

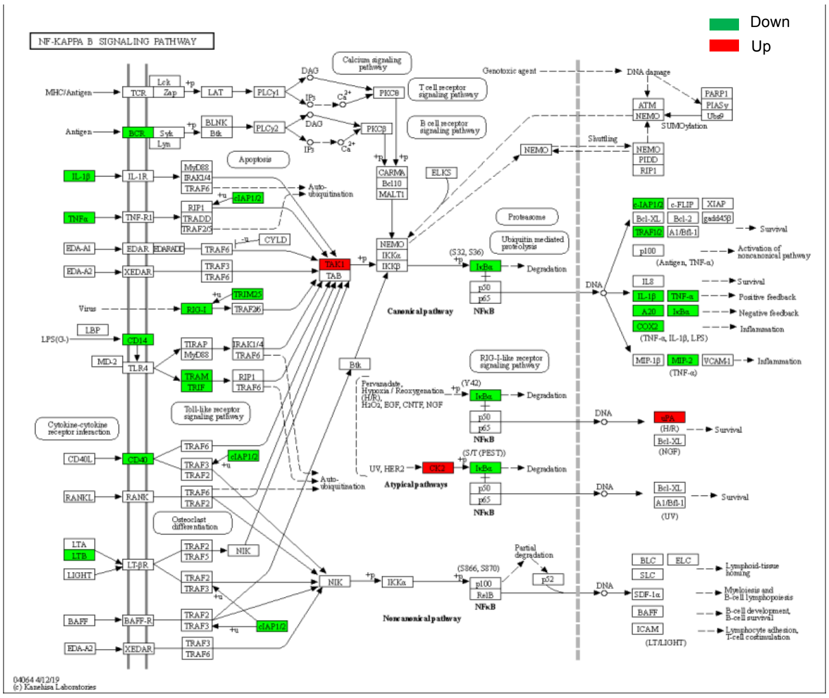

Figure S20. Gene ontology analysis and pathway enrichment analysis showed that NF-KAPPA B signaling pathway were activated.

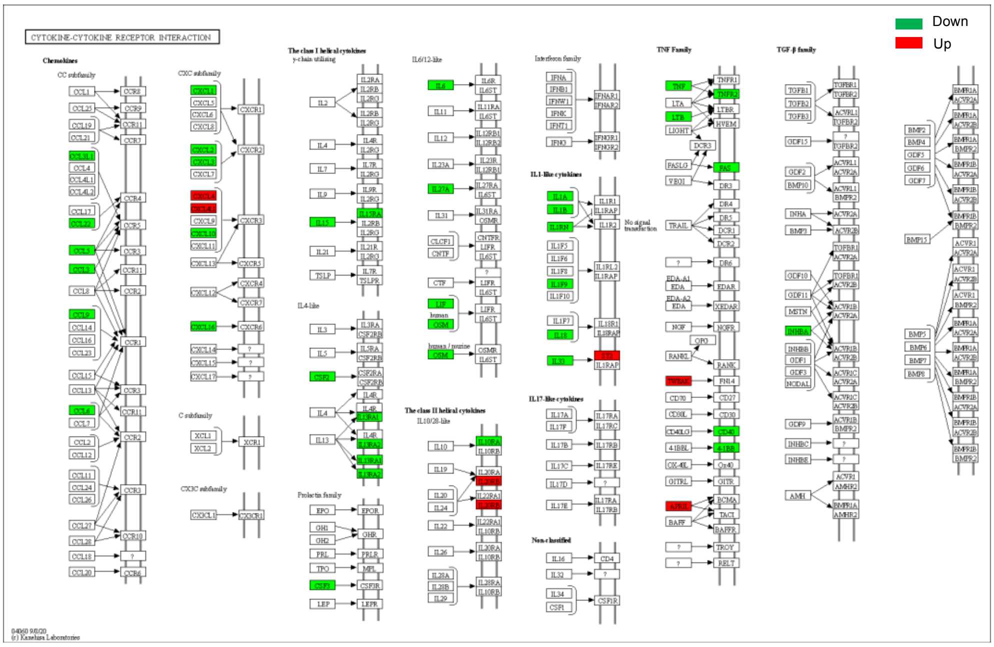

Figure S21. Gene ontology analysis and pathway enrichment analysis showed that Cytokine-Cytokine receptor interaction signaling pathway were activated.

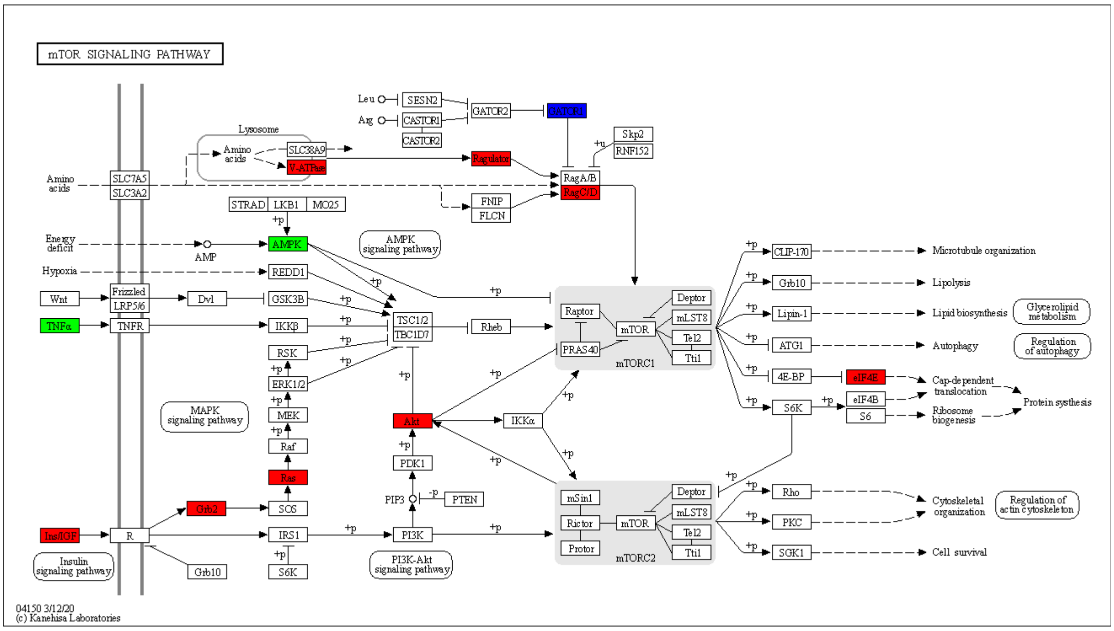

Figure S22. Gene ontology analysis and pathway enrichment analysis showed that mTOR signaling pathway were activated.

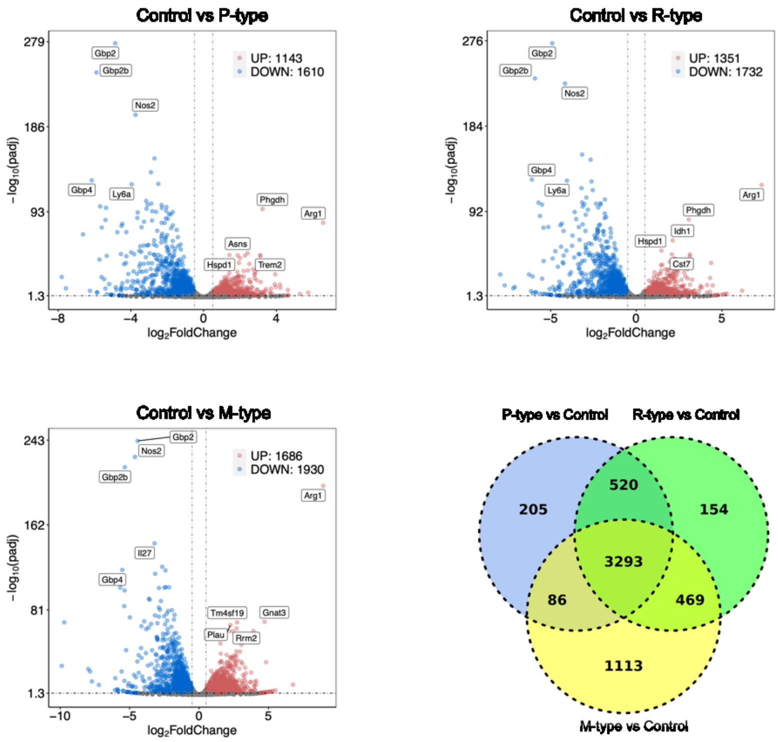

Figure S23. The volcano map showed the up-regulated expression of ARG1 in the *control* group, *P*-type, *R*-type and *M*-type groups. Venn diagram showed the number of different genes of *P*-type, *R*-type and *M*-type compared with the *control* group

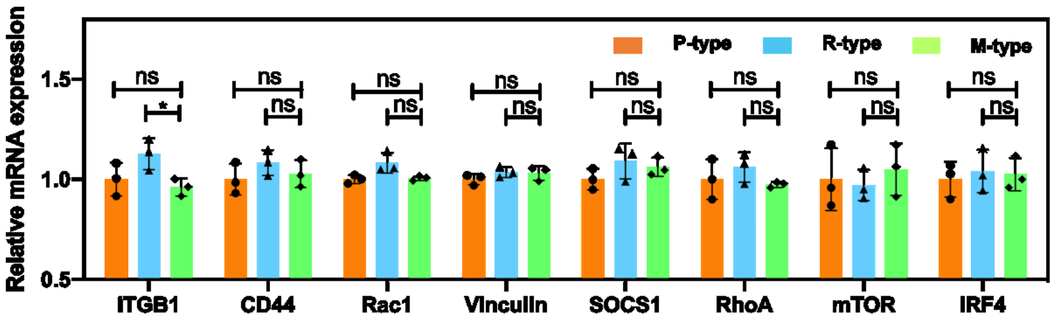

Figure S24. Y27632 antagonized the downstream signals of ROCK and BEZ235, and specifically blocked the downstream signals of PI3K and mTOR. RT-qPCR analysis showed that there were no significant differences in the expression of M2-related and mechanism pathway genes of macrophages in *M*-type matrixes compared with *P*- and *R*-type matrixes.

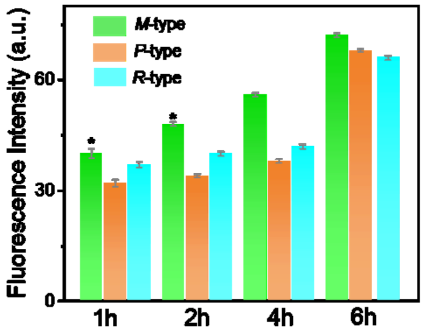

Figure S25. Quantitative analysis of immunofluorescence signal intensity of Fn adsorption after coincubation with each matrix.

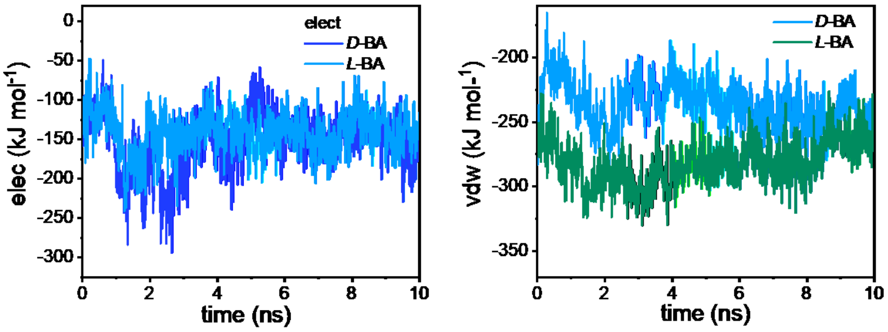

Figure S26. Computed average interaction energies showing the lower binding energy that is required during recognition between *D*-BA and FnIII7-10 than that between *L*-BA and FnIII7-10.

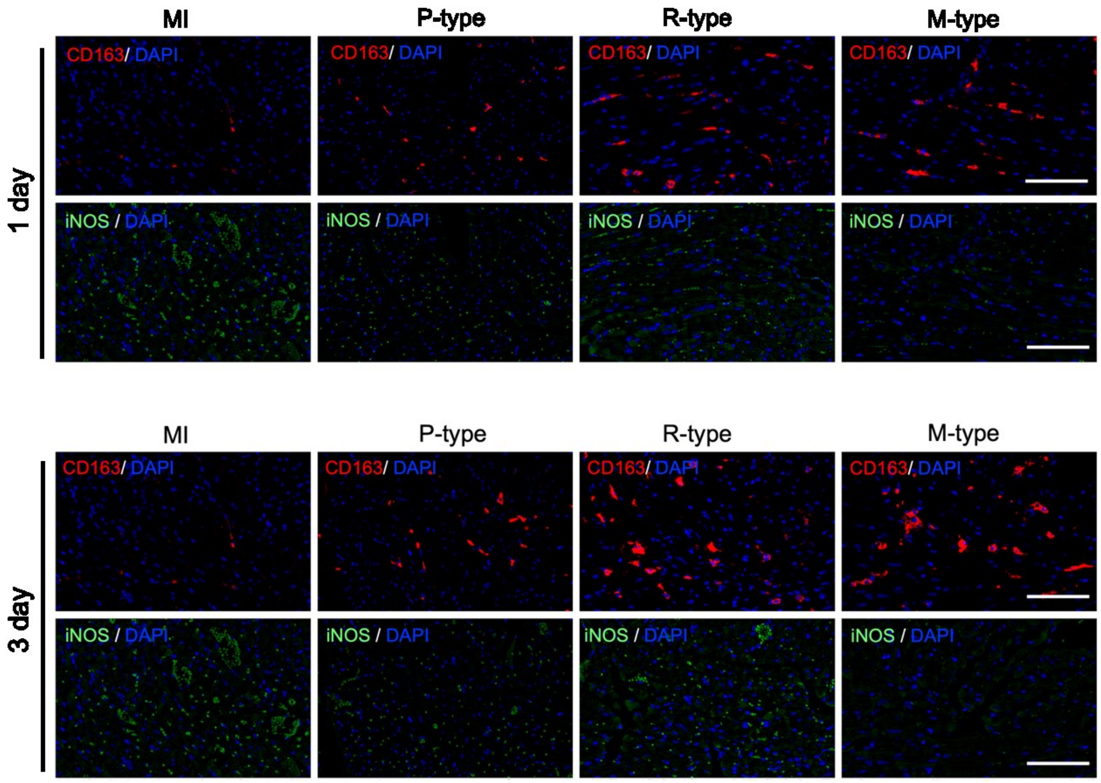

Figure S27. The expression levels of myocardial inflammatory factors iNOS and CD163 surface proteins in MI, *M*-type, *P*-type and *R*-type self-assembled matrices were evaluated by tissue immunofluorescence. scale :100 μm

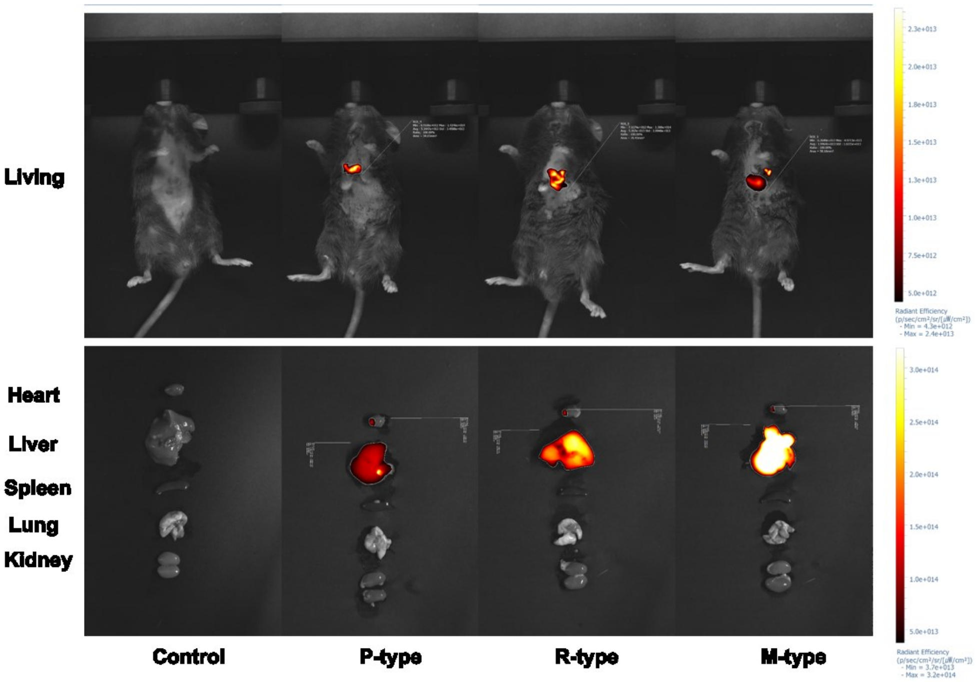

Figure S28. IR783B-NHS-doped chiral self-assembling matrixes were implanted into the MI region of rats, and effective accumulation and degradation of these substances in the infarcted heart were observed.

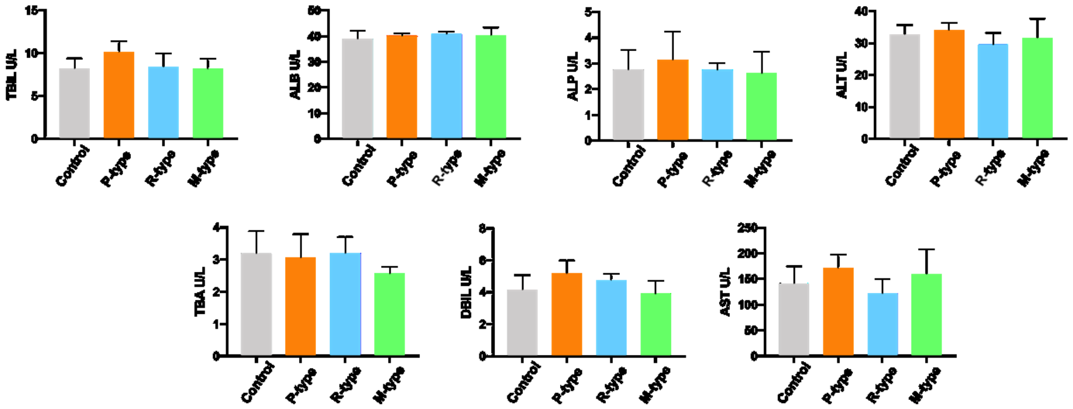

Figure S29. Effects of implanting chiral self-assembling matrixes on liver and kidney function in mice.

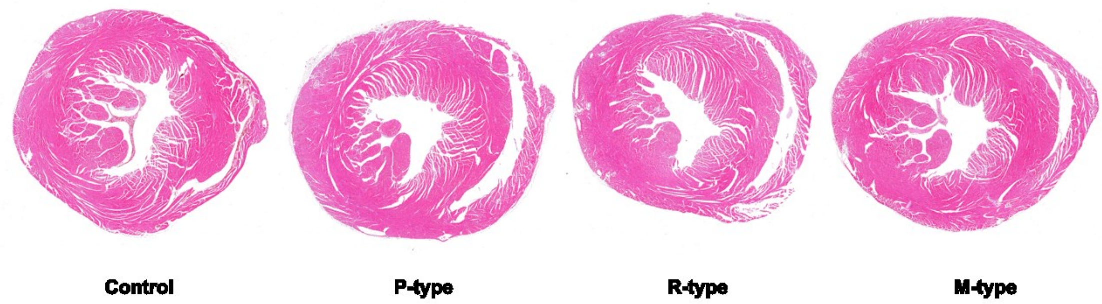

Figure S30. HE pathological diagnosis of heart tissue staining showed no significant toxicity in the hearts of mice implanted with different chiral self-assembling matrixes compared to the *control* group.

Table S2. Primer Sequences Used for RT-PCR Gene Expression Analysis

Gene	5′−3′	primers
TNF-α	Forward primer	CTGAACTTCGGGGTGATCGG
	Reverse primer	GGCTTGTCACTCGAATTTTGAGA
iNOS	Forward primer	GTTCTCAGCCCAACAATACAAGA
	Reverse primer	GTGGACGGGTCGATGTCAC
TLR9	Forward primer	ACAACTCTGACTTCGTCCACC
	Reverse primer	TCTGGGCTCAATGGTCATGTG

IL1-β	Forward primer	TACGGACCCCAAAAGATGA
	Reverse primer	TGCTGCTGCGAGATTTGAAG
IL-6	Forward primer	CTGCAAGAGACTTCCATCCAG
	Reverse primer	AGTGGTATAGACAGGTCTGTTGG
Ym1	Forward primer	AGAAGGGAGTTTCAAACCTGGT
	Reverse primer	GTCTTGCTCATGTGTGTAAGTGA
Fizz1	Forward primer	CTGGGTTCTCCACCTCTTCA
	Reverse primer	TGCTGGGATGACTGCTACTG
CD44	Forward primer	CTCAAGTGCGAACCAGGACAGTG
	Reverse primer	ATCAGAGCCAGTGCCAGGAGAG
RhoA	Forward primer	CCTCCTCGTCGCTTCCTCTCAG
	Reverse primer	GCTTCGCAGTGGCAGGCTAC
mTOR	Forward primer	ACCGTCCGCCTTCACAGATACC
	Reverse primer	GCAGTCCGTTCCTTCTCCTTCTTG

References

[1] Liu, J.; Yuan, F.; Ma, X.; Auphedeous, D. Y.; Zhao, C.; Liu, C.; Shen, C.; Feng, C. The Cooperative Effect of Both Molecular and Supramolecular Chirality on Cell Adhesion. *Angew. Chem. Int. Ed.* **2018**, *57*, 6475-6479.

[2] J. Liu, Y. Zhao, C. Zhao, X. Dou, X. Ma, S. Guan, Y. Jia, C. Feng, Hydrogen-bonding regulated supramolecular chirality with controllable biostability. *Nano Research*, **2022**, *15*, 2226-2234.

[3] Y. Sang, D. Yang, P. Duan, *Chem. Sci.*, **2019**, *10*, 2718-2724.

[4] J. Zhao, Y. Liu, A. Hao, P. Xing, *ACS Nano* **2020**, *14*, 2522-2532.
